# Mutant p53 Gain-of-Function: Role in Cancer Development, Progression, and Therapeutic Approaches

**DOI:** 10.3389/fcell.2020.607670

**Published:** 2021-02-11

**Authors:** Eduardo Alvarado-Ortiz, Karen Griselda de la Cruz-López, Jared Becerril-Rico, Miguel Angel Sarabia-Sánchez, Elizabeth Ortiz-Sánchez, Alejandro García-Carrancá

**Affiliations:** ^1^Programa de Posgrado en Ciencias Biológicas, Universidad Nacional Autónoma de México, Mexico City, Mexico; ^2^Subdirección de Investigación Básica, Instituto Nacional de Cancerología, Secretaría de Salud, Mexico City, Mexico; ^3^Doctorado en Ciencias Biomédicas, Instituto de Investigaciones Biomédicas, Universidad Nacional Autónoma de México, Mexico City, Mexico; ^4^Programa de Posgrado en Ciencias Bioquímicas, Departamento de Bioquímica, Facultad de Medicina, Universidad Nacional Autónoma de México, Mexico City, Mexico; ^5^Laboratorio de Virus and Cáncer, Unidad de Investigación Biomédica en Cáncer, Instituto de Investigaciones Biomédicas, Universidad Nacional Autónoma de México and Instituto Nacional de Cancerología, Secretaría de Salud, Mexico City, Mexico

**Keywords:** p53, gain of function, oncogenic pathways, metabolic reprogramming, stemness, chemo-resistance, immune evasion

## Abstract

Frequent p53 mutations (mutp53) not only abolish tumor suppressor capacities but confer various gain-of-function (GOF) activities that impacts molecules and pathways now regarded as central for tumor development and progression. Although the complete impact of GOF is still far from being fully understood, the effects on proliferation, migration, metabolic reprogramming, and immune evasion, among others, certainly constitute major driving forces for human tumors harboring them. In this review we discuss major molecular mechanisms driven by mutp53 GOF. We present novel mechanistic insights on their effects over key functional molecules and processes involved in cancer. We analyze new mechanistic insights impacting processes such as immune system evasion, metabolic reprogramming, and stemness. In particular, the increased lipogenic activity through the mevalonate pathway (MVA) and the alteration of metabolic homeostasis due to interactions between mutp53 and AMP-activated protein kinase (AMPK) and Sterol regulatory element-binding protein 1 (SREBP1) that impact anabolic pathways and favor metabolic reprograming. We address, in detail, the impact of mutp53 over metabolic reprogramming and the Warburg effect observed in cancer cells as a consequence, not only of loss-of-function of p53, but rather as an effect of GOF that is crucial for the imbalance between glycolysis and oxidative phosphorylation. Additionally, transcriptional activation of new targets, resulting from interaction of mutp53 with NF-kB, HIF-1α, or SREBP1, are presented and discussed. Finally, we discuss perspectives for targeting molecules and pathways involved in chemo-resistance of tumor cells resulting from mutp53 GOF. We discuss and stress the fact that the status of p53 currently constitutes one of the most relevant criteria to understand the role of autophagy as a survival mechanism in cancer, and propose new therapeutic approaches that could promote the reduction of GOF effects exercised by mutp53 in cancer.

## Introduction

Cancer is a complex set of diseases, all characterized by abnormal cell growth, unresponsive to normal cellular and tissue controls. It originates with wayward cells that once formed, grow, expand, and ultimately disseminate to other parts of the body, and in many cases, when not detected early, will ultimately kill their host. Cancer cells are characterized by dysregulated key elements and fundamental signaling pathways controlling proliferation, cell-death, interactions with the immune system, metabolic changes, and response to drugs, among the most relevant (Hanahan and Weinberg, 2011).

The behavior and status of p53 is fundamental for cancer development, progression, and for the fate of many cancer patients. p53 plays many important roles in cancer and is considered a master regulator of intracellular functions, such that it has appeared on the covers of the most prominent science journals, like Science and, and has been awarded titles such as “*the guardian of the genome”* (Finlay et al., 1989; Soussi et al., 1990; Yeargin and Haas, 1995).

**Figure 1:**
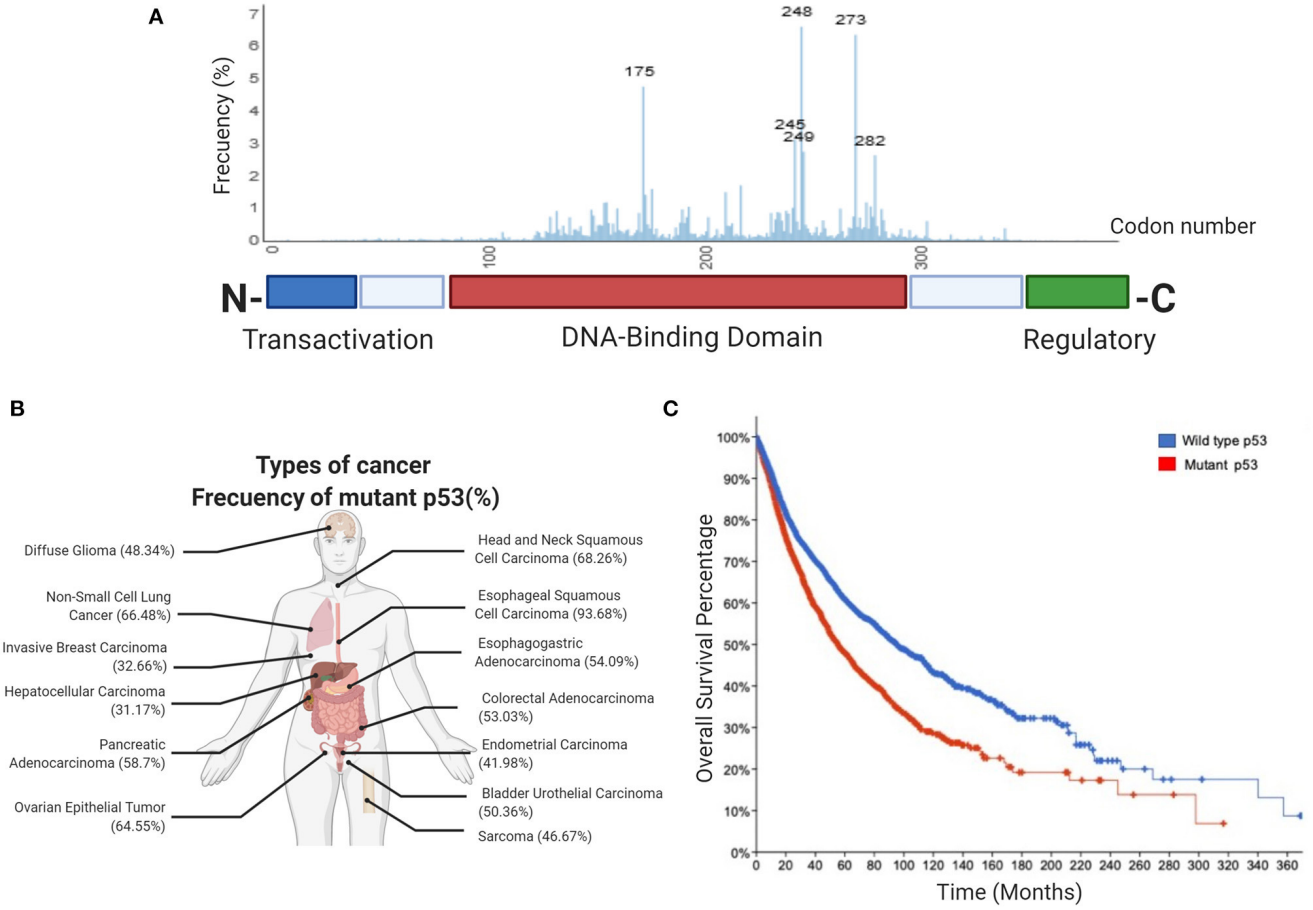
Frequency of p53 mutations in human cancers. **(A)** Schematic picture showing the domain structure of the p53 protein, including the transactivation domain, DNA-binding domain and regulatory domain. The aligned graphs indicate the relative frequency of mutations across different domains of p53. p53 mutations are most frequently found in the DNA-binding domain, according to the IARC TP53 database. **(B)** Percentage frequency of TP53 gene alterations in different types of cancer. The data were obtained from TCGA PanCancer Atlas using a combined study (*n* = 10,967). **(C)** Overall survival for human cancer patients (*N* = 10,953 patients from 32 studies) with mutp53 (red line) or wild type p53 (blue line). The graph was analyzed and obtained from cBioportal.

It is well established that altogether, around half of all human tumors exhibit alterations in *TP53* alleles, either by inactivation, loss or, importantly, mutations. Tumor cells containing mutant alleles of this gene generate mutant versions of the protein that, remarkably, mainly affect amino acids located within the DNA binding domain (DBD) (Figure 1). These mutant versions of p53 not only lead to loss of normal functions but surprisingly, confer mutant proteins with new abilities that provide cancer cells with key gain-of-function activities (GOF's).

Recently, the mechanisms and effects of these mutant alleles have been shown to affect key biological processes associated with cancer progression, invasion, metabolic reprograming, and interactions with the immune system. The study of such effects on central processes including proliferation, migration, generation of an inflammatory microenvironment, metabolic reprogramming, stem-cell restricted characteristics, and pharmacological resistance, has gained much attention. Although these processes are central for cancer, the molecular mechanisms involved and the precise targets acted upon by mutp53 GOF's, are only recently being elucidated.

Understanding the mechanisms involved and the effects of mutp53 GOF will be vital to better combat pharmacological resistance of cancer cells that harbor mutp53, and to design effective therapies based on p53 status in different types of cancer.

This review aims to integrate novel data on mechanisms and targets involved in the effects of mutp53 GOF's, stressing current knowledge of the central pathways involved.

## Discovery

The product of the *TP53* gene was first observed in the 1970's by several groups when studying cellular transformation of rodent cells induced by a simian virus called SV40. Transformation was observed when non-permissive cells were infected or rodents were injected with SV40, leading to tumor development and a strong host immune response against a viral protein called T antigen (TAg). Several groups used a monoclonal antibody to immunoprecipitate TAg from transformed cells. Although they observed a 53–54 kDa protein in polyacrylamide gels, the nature of this protein and its specific association with TAg was not evident (Chang et al., 1979). Simple experiments revealed this as a cellular protein specifically associated with TAg and two seminal papers suggested that this protein, named p53, represented a key element for viral transformation (Lane and Crawford, 1979; Linzer and Levine, 1979). A few years later, when a murine cDNA coding for TP53 was cloned and shown to transform fibroblasts in culture, it was stated that *TP53* was “just another oncogene” and was recognized as such for a long time (Oren and Levine, 1983; Parada et al., 1984).

Rearrangements of the *TP53* gene were found in several human tumors and more importantly, loss-of-heterozygosity, a characteristic of tumor suppressor genes, was commonly observed (Masuda et al., 1987). Although these different lines of evidence strongly suggested that *TP53* was not just another oncogene, at the time, few envisioned that it would emerge as prototype of all human tumor suppressor genes so far identified.

Finally, when the effect of that same human gene on transformed cells was studied, it clearly showed its nature as a tumor suppressor gene.

## Canonical Functions

**Figure 2:**
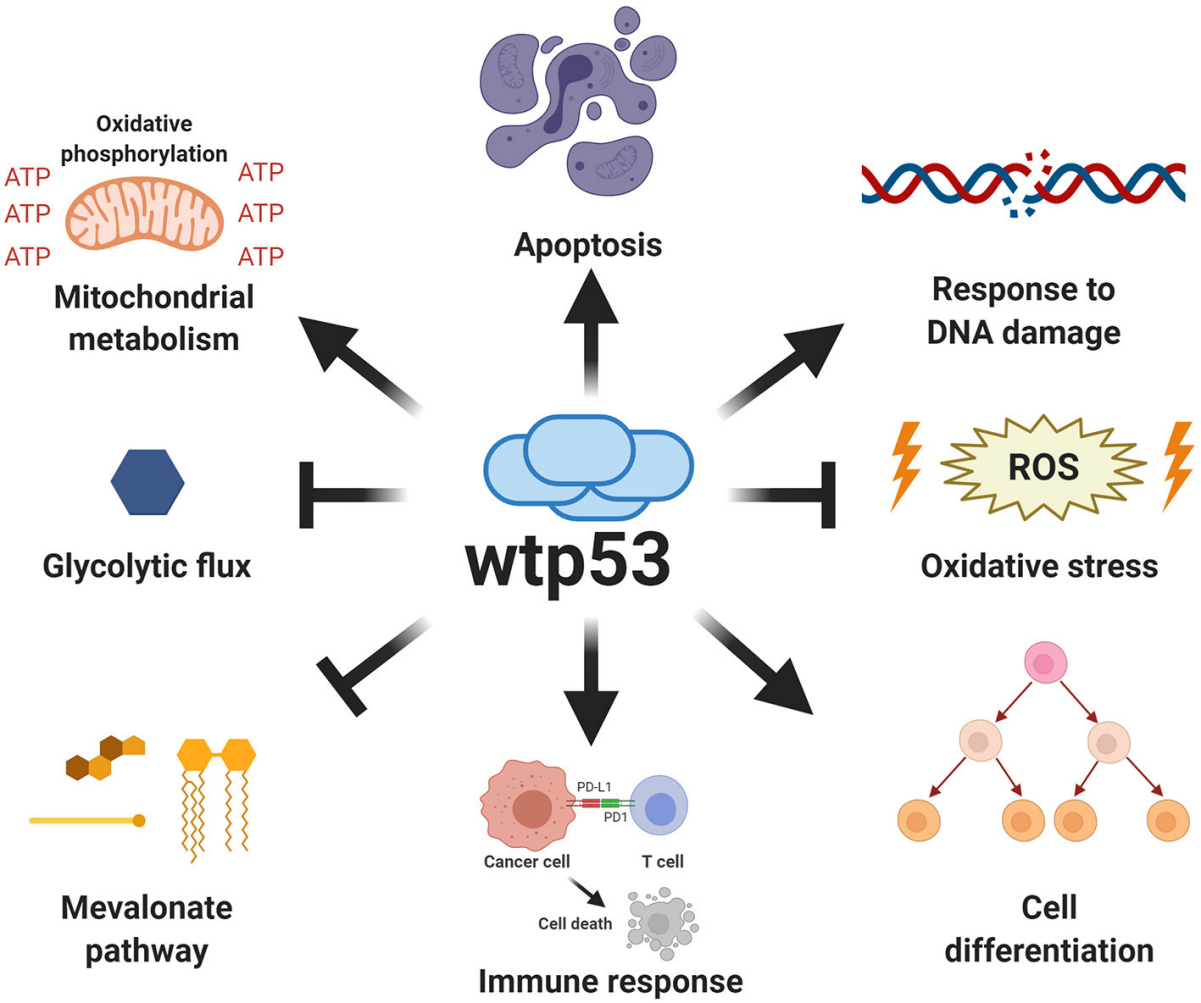
Canonical functions of wild type p53. Wild type p53 is a major tumor suppressor whose functions are critical for protection against cancer. The canonical functions of wild type p53 include the induction of apoptosis, regulation of oxidative metabolism, and inhibition of glycolytic flux, as well as the response to DNA damage, increased antioxidant capabilities, regulation of immune response and differentiation processes.

A vast number of signals promote several p53-mediated functions, including cellular stress, DNA damage, hypoxia, nutritional stress, as well as differentiation signals. Activation of p53 (referred to as wtp53) drives a plethora of signals that fire different fundamental responses such as cell-cycle arrest, apoptosis, senescence, regulation of cellular energy metabolism, antioxidant defense, and immune system regulation (Figure 2). Relevant target genes of the p53 transcription factor encode proteins, such as p21 and p27 that induce cell-cycle arrest, PAI1 and CDKN1b involved in inducing senescence, PUMA, BAX, and NOXA involved in apoptosis, or TIGAR and GLS2 for metabolic changes, among others (Kastenhuber and Lowe, 2017; Simabuco et al., 2018).

One of best described roles for p53 is in the response to DNA damage. Acting as a classic transcription factor, p53 induces the expression of p21, which in turn inhibits CDKs (cyclin-dependent kinases), resulting in cell-cycle arrest (Deng et al., 1995).

Another function involves regulation of oxidative phosphorylation and mitochondrial respiration through the induction of COX (cyclooxygenase) and GLS2 (glutaminase 2) expression. GLS2 is an enzyme involved in deamination of glutamine, allowing production of α-ketoglutarate, an intermediary metabolite of the TCA cycle (tricarboxylic acid cycle) (Hu et al., 2010). Antioxidant defense mechanisms modulated by p53 include increasing levels of GST (glutathione S-transferase), an important enzyme implicated in avoiding the deleterious effects of ROS (reactive oxygen species) (Puzio-Kuter, 2011).

Furthermore, key functions of p53 include regulation of immune system interactions (Blagih et al., 2020). The main effector that allows p53 to orchestrate these interactions is NF-kB (Komarova et al., 2005). NF-kB constitutes a key transcription factor acting as one of the main regulators of pro-inflammatory activity in many immune responses. Its activation in leukocytes promotes the expression of pro-inflammatory cytokines (IL-1, IL-2, IL-6, TNF-α), chemokines (CXCL1, CXCL10, MCP-1), adhesion molecules (ICAM- 1, VCAM-1, ECAM-1), and anti-apoptotic factors (BCL-2, c-Flip, survivin) (Liu T. et al., 2017). NF-kB activation is normally suppressed by binding to its inhibitors, a family of proteins known as IkB.

Following a pro-inflammatory stimulus, IKKs (IkB kinases) are activated and phosphorylate IkB inhibitors, favoring their degradation, thus enabling NF-kB transcriptional activity (Dresselhaus and Meffert, 2019). NF-kB regulates recruitment, survival, proliferation, activation, and differentiation of leukocytes, in response to antigen recognition or activating signals (Liu T. et al., 2017). Different reports have confirmed the relationship between p53 and NF-kB, showing that wtp53 negatively controls the expression and activity of NF-kB (Gudkov et al., 2011; Blagih et al., 2020). This evidence indicates that regulation of wtp53 over the immune system is mainly due to a mutual repression between wtp53 and NF-kB that promotes an anti-inflammatory microenvironment.

However, wtp53 immune regulatory functions are not limited to repression of pro-inflammatory responses. For instance, MHC-I (Major Histocompatibility Complex Class I) is positively regulated by wtp53, promoting T cell recognition. Thus, alterations in *TP53* also lead to deficient immune system responses (Wang B. et al., 2013; Blagih et al., 2020).

Understanding wtp53 actions, it is possible to establish the effects derived from mutp53 GOF, being an important criterion in biological processes ranging from proliferation, migration, metabolism reprogramming, immune evasion, state of differentiation, and chemoresistance, which are explained in the following sections.

## Impact of Mutp53 Gain-of-Function

Although it is obvious that mutations in the *TP53* gene should result in loss of canonical functions, in the last years it has become evident that the most common mutant alleles acquire new functions that fuel tumor progression.

Most common GOF mutations in *TP53* are located within the DBD, as have been observed in many different types of human solid tumors. Conversely, p53 mutations located in the regulatory or tetramerization domains are less frequent. Common mutants have single missense mutations leading to single amino acid substitutions at key “hotspots” that preponderantly include residues 175, 248, and 273, as shown in Figure 1A. These high-frequency mutants represent non-random “hotspots” and correlate with poor cancer-free survival (Figures 1B,C).

There are two main types of mutant “hotspot” sites: contact mutants (R273H, R248Q, and R248W) and conformational mutants (R175H, G245S, R249S, and R282H), both affecting the DBD. Contact mutants generally produce structural changes in the p53 protein that directly affect DNA binding, while conformational mutants generate structural changes related with protein folding, both types of mutants have shown GOF activities (Kim and Lozano, 2018).

The effect of mutp53 is reflected on tumorigenic ability. Particularly, germline p53 mutants are mainly associated to Li-Fraumeni Syndrome, in which patients are more likely to develop tumors (Lang et al., 2004; Olive et al., 2004). Additionally, the effect of mutp53 has been described in transgenic mice able to express mutp53 in a tissue-specific manner (Wijnhoven et al., 2005). This versatility made it possible to determine that mutp53 can promote metastasis in a genetic context where mutant versions of other proteins, such as oncogenic Ras, are present (Morton et al., 2010). This evidence suggests that multiple oncogenic effects drive tumorigenesis and metastasis. In the case of colorectal cancer, even though loss of wtp53 improves the oncogenic capability of cancer cells through LOH, the presence of mutp53 in both alleles seems to be necessary to drive tumorigenesis (Nakayama et al., 2020). Moreover, intracellular accumulation of mutant versions of p53 increases the effects of signaling pathways affected by new GOF activities (Pfister and Prives, 2017).

### Nuclear Effects of mutp53

A consensus response element (RE) is required for wtp53 to bind and regulate expression of target genes. However, the mutations in the p53 DBD lead to disruption in the ability to bind this RE. Importantly, mutp53 can bind to novel non-canonical DNA binding sites of several genes and thus, can positively or negatively regulate the expression of genes associated with malignancy (Kim and Deppert, 2007). This versatility of the nuclear effects of mutp53 has just recently been described, and it becomes relevant for the GOF associated with mutp53 (Göhler et al., 2005).

Even though DNA-binding of mutp53 does not depend on particular sequences, the mechanisms of mutp53-mediated transcription can be direct or indirect. Employing electromobility shift assays (EMSAS) and confocal fluorescence lifetime microscopy it was shown that mutp53 (248P and 245S) can bind specifically and selectively to non-B DNA. Binding does not require the presence of specific sequence motifs but indeed requires both the DBD and an intact p53 C-terminal regulatory domain (CRD). This mode of binding was termed as “DNA structure selective binding” (DSSB) (Göhler et al., 2005). In support of this, several mutant versions of p53 have been shown to bind preferentially to supercoiled DNA and by luciferase reporter assay demonstrated that the DNA topology influences p53 regulation of BAX and MSP/MST1 promoters (Brázdová et al., 2013). Moreover, it was shown that mutp53 binds efficiently to nonlinear DNA, and this can be increased by apurinic/apyrimidinic endonuclease 1/redox factor-1 (APE1) that stimulate DNA biding activity of numerous transcription factors in a redox-dependent manner (Cun et al., 2014).

For instance, mutp53 can bind to DNA on non-canonical sites from non-linear conformations (Göhler et al., 2005). It is important to mention that there are physical interactions of mutp53 with remodeling complexes that cause changes in the transcriptome, conferring plasticity in gene expression patterns. In this sense, it has been reported that binding of mutp53 to particular motifs on non-B DNA conformations, confers stability to mutp53, but also makes it more selective for modifying the activity of both transcription factors and chromatin remodelers (Göhler et al., 2005; Freed-Pastor and Prives, 2012). For instance, mutp53 has been related with the activity of the SWI/SNF chromatin complex, which increases histone modifications promoting an “open” state of chromatin, influencing the global transcriptome and the expression of cancer-related genes. The fact is that >40% of gene expression related to mutp53 can be explained by the effect on the SWI/SNF complex (Pfister et al., 2015).

Moreover, overlap in the DNA binding sequence patterns was observed through which mutp53 can act on response elements of other transcription factors, modulating gene expression (Agostino et al., 2006). For example, mutp53 can regulate gene expression through physical binding to p53 family members with tumor suppressor capacity such as p63 and p73, and modify their transcriptional activity (Ferraiuolo et al., 2016). Recently, it was shown that mutp53 interacts with the intracellular domain of Notch1 to abrogate p63/p73 mediated repression of HES1 and ECM, promoting lymphomagenesis (Zhang et al., 2019). A more complete picture has been shown with newer evidence pointing out that mutp53 can act as a nuclear repressive factor to downregulate pro-apoptotic responses, such as expression of the CD95 gene (Fas receptor), which is involved on apoptosis induction (Zalcenstein et al., 2003). Moreover, mutp53 is able to both positively and negatively regulate the activity of a variety of transcription factors, such as ETS2, NF-kB, HIF-1α, SMAD, SREBP, or NF-Y (Kim and Lozano, 2018). For instance, mutp53 may directly cooperate with YAP1 (Yes-associated protein) and favor the transcriptional activity of NF-Y on proliferation-related genes, suggesting that mutp53 may act as a transcription cofactor to enhance GOF (Di Agostino et al., 2016). The nuclear effect of mutp53 is an interesting field of study that is not well clarified.

### Proliferation, Invasion and Metastasis

**Figure 3:**
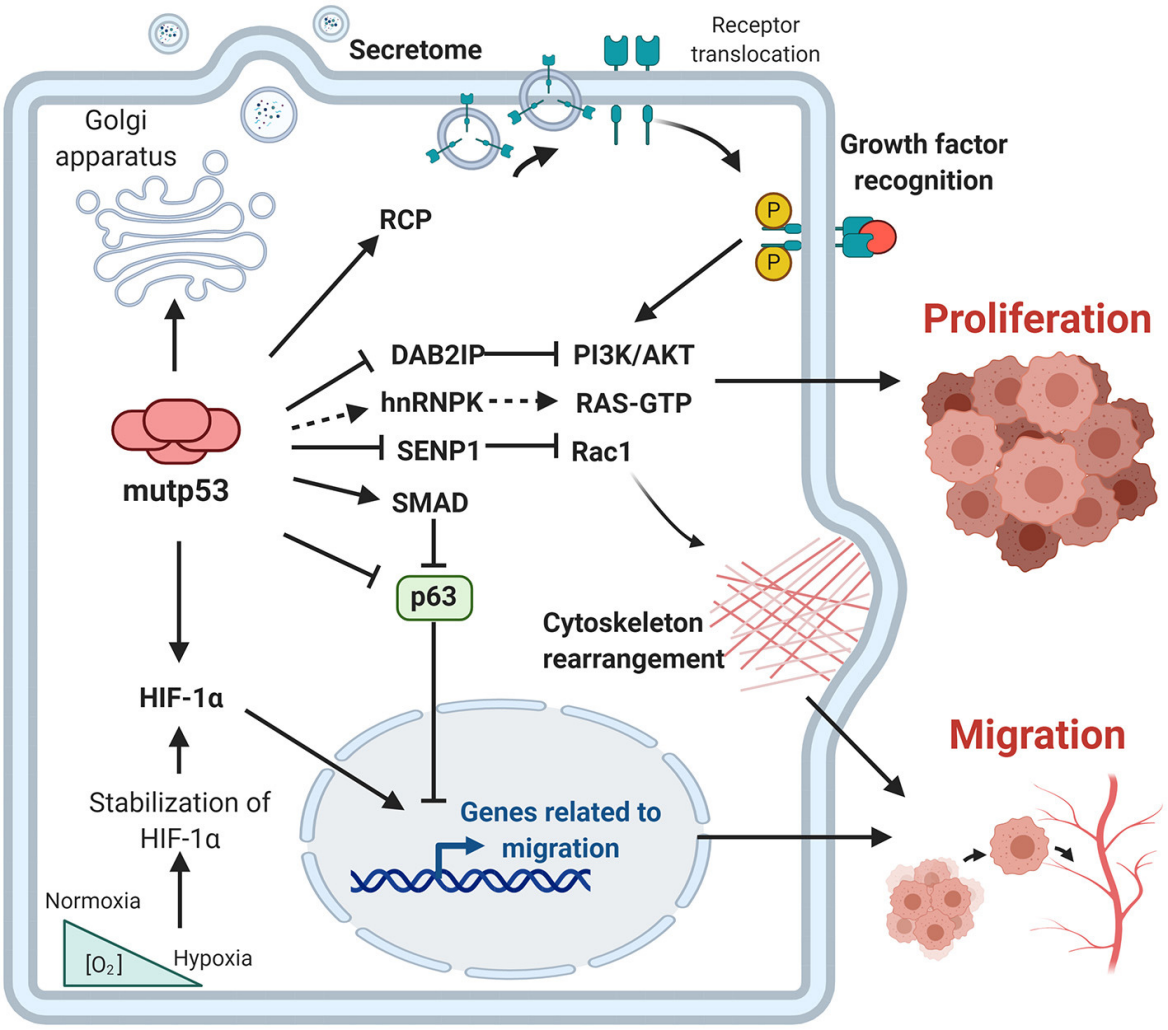
Gain-of-function of mutant p53 over proliferation, invasion and metastasis. The principal GOF activities of mutant p53 have nuclear and non-nuclear effects. The nuclear effects involve binding to transcription factors such as HIF-1α or p63 and p73, while the non-nuclear effects are regulation of intracellular proteins, such as RCP, implicated in receptor translocation, DAB2IP scaffold protein implicated in the PI3K/AKT pathway, or SENP1, a protease related to Rac1 activity.

In the last decade, important contributions have allowed us to better understand the mechanisms involved and the impact of mutp53 GOF on cell proliferation, invasion and metastasis. The particular importance of mutp53 is in promoting proliferation, invasion and metastatic potential through its effect on the endosomal pathway, leading to recycling of receptors and integrins. For instance, overexpression of mutp53 has been shown to increase translocation of EGFR (Epidermal Growth Factor Receptor) and α5β1 integrin on the surface of cell membranes. This translocation is dependent on interaction with RCP (Rab-coupling protein) (Muller et al., 2009). As a consequence, many of the intracellular pathways associated with the regulation of endosomal pathways, including PI3K/AKT or MAPK cell signaling pathways, are activated by mutp53 (Figure 3).

Additionally, it has been reported that the R273H mutant binds and represses the promoter region of miR-27a, a microRNA that negatively regulates the EGFR transcript. This reinforces the evidence that the presence of mutp53 can favor the activity of signaling pathways related to EGFR, as well as the downstream signaling mechanisms. Gastric cancer tumor samples corroborate this effect, showing reduced expression of miR-27a compared to normal tissue (Wang W. et al., 2013).

Other effects attributed to mutp53 are mediated by the regulation of the PI3K/AKT pathway through binding DAB2IP (DAB2-interacting protein). DAB2IP is a scaffold protein that binds to and inactivates p85-PI3K, impairing its repressive functions over PI3K, promoting the intracellular effects of AKT1. Thus, growth factors, such as insulin, increase proliferation in prostate and breast cancer (Valentino et al., 2017).

Recent findings reveal that mutp53 (R175H) exacerbate the oncogenic response of K-Ras. The active state of K-Ras (G12C) is related to its GTP-binding form, while the GTPase-activating proteins (GAPs) can favor the GDP inactive form. Importantly, K-Ras activity is not enough to promote tumorigenic capacities in models such as pancreatic ductal adenocarcinoma, since the K-Ras mutant form cannot maintain the GTP-bound state. Nevertheless, mutp53 can regulate the splicing of GAPs through RNA-binding protein hnRNPK. The activity of mutp53 favors the expression of GAP isoforms that cannot bind to Ras, abrogating the ability to decrease its activity and supporting the oncogenic effect of K-Ras. This mechanism reveals a synergism between K-Ras and mutp53 through spliceosome effects, supporting malignant progression through the effect of multiple oncogenes like K-Ras (Escobar-Hoyos et al., 2020).

Additionally, it was found that mutp53 inhibits apoptosis associated with mitochondria and confers resistance to anoikis (Tan et al., 2015), a type of cell death related with loss of contact with the extracellular matrix or neighboring cells. Apoptosis associated with mitochondria requires the dissociation of the proapoptotic protein BIM from the antiapoptotic protein BCL-XL. However, the presence of the p53-R273H mutant suppresses BMF (BCL2-modifying factor) expression, which is a protein that induces cellular anoikis and apoptosis by reducing the interaction between BIM and BCL-XL. Knockdown of endogenous mutp53 restores sensitivity to apoptosis, highlighting the importance of mutp53 not only in cellular proliferation, but in cell survival of lung, colon, and breast cancer cell lines (Tan et al., 2015).

Considering receptor recycling generated by RCP, it has been suggested that cellular scattering could be attributed to HGF (Hepatocyte growth factor) as well as the presence of mutp53. Both elements potentiate MET (HGF receptor) activity, increasing its phosphorylation. The main biological responses are cytoskeletal changes that allow cell motility (Jo et al., 2000). The presence of mutp53 not only exacerbates migratory abilities through the MET receptor, but also promotes inhibition of p63, a key transcription factor regulating expression of anti-metastatic genes, evidencing that the effects of mutp53 are not limited to a particular mechanism (Muller et al., 2009, 2013).

Moreover, in a model of endometrial cancer mutp53 can promote EMT (epithelial-mesenchymal transition). Studies on miR130b, have determined that mutp53 is partially responsible for promoting an invasive phenotype through binding of mutp53 to the promoter region of this microRNA, thereby repressing its transcription. MiR130b inhibits Zeb1 expression, a transcription factor involved in regulating the expression of EMT markers. Thus, mutp53 represses transcription of miR130b and increases transcription of Zeb1, favoring invasion (Dong et al., 2013).

Studies employing immunoprecipitation assays have shown that mutp53 interacts with the small GTPase Rac1 and inhibits its interaction with SUMO-specific protease 1 (SENP1) favoring an active state of Rac1 (Yue et al., 2017). Thus, Rac1 activation is an important mechanism by which mutant p53 GOF promotes tumor metastasis.

Furthermore, accumulation of versions of mutant p53 seems to favor GOF and the chaperone machinery mediated by Hsp90 partially explains mutp53 stabilization. Furthermore, Hsp90 can be secreted by cancer cells, specifically those with mutp53 (R175H), influencing ECM degradation as well as migratory capacities. This effect is explained by the mutp53/RCP axis, favoring colonization to distant sites, such as the lung. Importantly, targeting the extracellular effect of Hsp90 decreases the invasive capacities of mutp53 cancer cells (Zhang S. et al., 2020). This evidence opens new avenues for the use of Hsp90 inhibitors in patients with mutp53.

Part of the effects of mutp53 over migration rely on other members of the p53 family, which include p63 and p73 transcription factors. These transcription factors share a conserved DBD that allows them to regulate the expression of a common pool of genes that are crucial for preventing tumorigenesis. Although p63 and p73 form homo and hetero tetramers, neither can bind to wtp53. Conversely, it has been found that several mutp53 versions can interact with both p63 and p73, and inhibit their transcriptional activity. It was shown that the recombinant core domain of some mutp53 proteins, but not wtp53, binds and inhibits p63 by masking its DBD (Gaiddon et al., 2001; Strano et al., 2001).

It is well accepted that GOF of mutp53 includes the ability to sequester the transactivation (TA) domain isoform of p63 and inhibit its interaction with its canonical DNA response element, thereby disrupting its downstream anti-metastatic transcriptional networks (Strano et al., 2001). Additionally, Neilsen et al. (2011) demonstrated that mutp53 GOF activities aberrantly alter the gene expression pattern of cancer cells to promote oncogenesis, involving a collaborative approach with p63 transcription factor. They show that mutp53 uses p63 as a molecular chaperone to bind to the promoter of target genes causing reprograming of the transcriptome. These genes are mainly associated with cellular invasion. These studies show that mutp53 can induce the secretion of pro-invasive factors to the surrounding microenvironment (Neilsen et al., 2011).

Importantly, the effect of mutp53 over p63 is decisive for signaling pathways like TGF-β (Transforming Growth Factor β), to determine whether they act as tumor suppressors or promoters of cellular migration and metastasis. Extracellular TGF-β receptor ligands exert their actions through Smad 2/3 transcription factors. Under non-cancerous contexts, they act as cell growth suppressors, but in the presence of mutp53 they improve migration ability, highlighting the pleiotropic relevance of TGF-β in cancer. Interestingly, it was shown that p63 is functionally inactivated when complexed with mutp53 and Smad in the presence of TGF-β ligands, this being critical for supporting metastasis. Moreover, this process is dependent on mutp53 N-terminal phosphorylation by oncogenic Ras. Mechanistically, mutp53 and Smad intercept p63 to form a ternary complex in which the p63 transcriptional functions are antagonized, offering an interesting explanation for the migratory effects induced by TGF-β (Adorno et al., 2009). Additionally, other reports revealed that mutp53 binds to the MH2 domain of Smad3, promoting a decrease in canonical TGF-β pathway signaling (Ji et al., 2015).

Moreover, studies have shown that part of the functions of mutp53 are involved with adapting to a hypoxic microenvironment, which favors an invasive phenotype. In this sense, there is dual participation between mutp53/HIF-1α, which allows for increased expression of extracellular matrix proteins, such as VIIa1 collagen and laminin-γ2, promoting an invasive phenotype in non-small cell lung cancer (Kamat et al., 2007; Amelio et al., 2018). Other reports support this premise, since it has been shown that there is an increase in tumor vascularization, as reflected by VEGF expression, as well as an increase of ROS in cell lines with mutp53 status (Khromova et al., 2009).

This is reinforced by evidence suggesting that the expression of pro-angiogenic isoforms of VEGF, but not anti-angiogenic isoforms, seem to depend on the interaction between mutp53 and the ribonucleoprotein complex composed by MALAT1 lncRNA, SRSF1, and ID4, favoring splicing of VEGF pro-angiogenic isoforms. This being an important axis in breast cancer cells (Pruszko et al., 2017). Recently, it has been reported that the effect of mutp53 reflects on morphological alterations of the Golgi apparatus which lead to alteration of the secretome of cancer cells, promoting release of soluble factors into tumoral microenvironment, including VEGF. From a mechanistic overview, this effect is explained by the dual action of mutp53 and HIF-1α through miR-30d, under both hypoxia and normoxia conditions. The secretome alteration promoted by mutp53 exercises important effects on primary and distant sites during carcinogenesis (Capaci et al., 2020).

### Metabolic Reprogramming

Hyperactivation of oncogenic pathways directly regulates the metabolic pathways that support tumor growth. Interestingly, mutp53 has been shown to enhance the Warburg effect, a process characterized by an increase in glucose uptake and lactate secretion even in the presence of oxygen (Levine and Puzio-Kuter, 2010; Eriksson et al., 2017). It was shown that mutp53 increases translocation of the glucose transporter GLUT1, without affecting total protein levels, favoring glucose uptake. The mechanistic effect is explained by an upregulation of the RhoA pathway. RhoA is a protein involved in different intracellular pathways like the activation of the effectors, ROCK1/2, which has been demonstrated to improve the distribution of transporters to the cell membrane in different cell types. Impairment at different points of the mutp53/RhoA/ROCK axis promotes an important decrease in glycolytic flux in different types of cancer cell lines (Zhang et al., 2013). This constitutes one of the first reports that explains how the presence of mutp53 favors the Warburg effect. Moreover, other reports support this evidence, showing that wtp53 antagonizes the Warburg effect and favors oxidative phosphorylation (Zhou et al., 2014; Hernández-Reséndiz et al., 2015).

Some authors have recently focused on the regulation of the mevalonate (MVA) pathway implicated in lipid metabolism and posttranslational modifications related to the malignant process. The biological effects of mutp53 on the regulation of the MVA pathway explain various cellular processes ranging from proliferation to fitness, or the regulation of the tumor microenvironment, all of them with functional relevance for tumorigenesis (Mullen et al., 2016; Ingallina et al., 2018).

Generation of MVA requires sequential action of enzymes, among which HMGCR constitutes a key element. Transcriptional regulation of HMGCR is controlled by SREBP (Sterol Regulatory Element-Binding Protein), which recognizes sterol-response elements on its promoter region (Mullen et al., 2016). Importantly, simultaneous binding of mutp53 and SREBP has been demonstrated on promoter regions recognized by SREBP using ChIP assays of genes implicated in the MVA pathway, including HMGCR, in breast cancer (Freed-Pastor et al., 2012). Thus, a great number of small GTPases, such as Rho and Ras, whose post-translational modifications are regulated downstream of MVA pathway can be increased by mutp53 (Freed-Pastor et al., 2012; Parrales et al., 2016).

**Figure 4:**
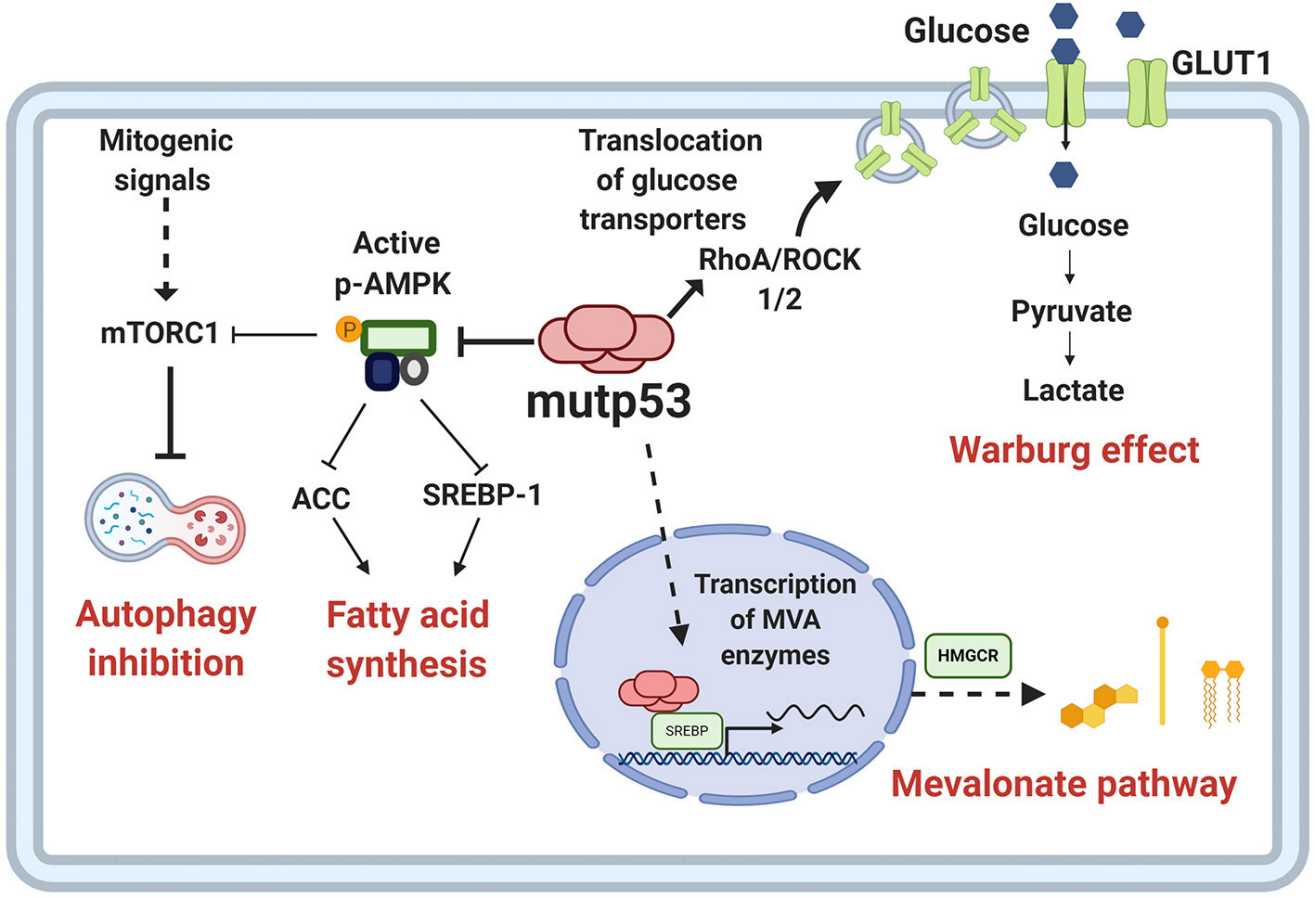
Metabolic reprogramming by mutp53. Mutp53 GOF activities are involved in different critical points of tumor metabolism. Mutp53 favors glucose uptake and hence the Warburg effect through membrane translocation of the glucose transporter, GLUT1, via the RhoA/ROCK1/2 axis. Moreover, mutp53 can induce the Warburg effect by directly inhibiting AMP-activated protein kinase (AMPK), a major cellular energy sensor and a master regulator of metabolic homeostasis. AMPK downregulates fatty acid synthesis by inhibiting transcription factor sterol regulatory element-binding protein 1 (SREBP1). Mutp53 increases the activity of SREBP1, a master regulator of fatty acids and cholesterol biosynthesis, and thus, the mevalonate (MVA) pathway.

The increased activation of anabolic pathways is an essential characteristic of cancer cells because they enable production of the macromolecules required for replicative cell division and tumor growth. One of the proposed mechanisms through which mutp53 favors the activation of anabolic pathways relies on AMPK inhibition, contrary to wtp53, which increases AMPK activity (Feng et al., 2007). AMPK is a Ser/Thr kinase activated by an increase in AMP levels, caused by energy stress (Zhou et al., 2014). AMPK decreases anabolic pathways such as fatty acid synthesis and protein synthesis, and promotes catabolic pathways including oxidation of fatty acids and autophagy. Mutp53 (R175H) can bind directly to the AMPKα subunit, thereby inhibiting activation of AMPK by upstream kinases. The consequences of this interaction, besides AMPK inhibition, is that the downstream targets of this kinase are not being regulated, and therefore, there is an increase in glycolytic flux, as shown in Figure 4 (Zhou et al., 2014).

Metabolic alteration and GOF related to mutp53 in cancer cells can increase the levels of reactive oxygen species (ROS). Moreover, the presence of mutp53 decreases NRF2 (Nuclear factor erythroid 2–related factor 2) activity and glutathione synthesis, promoting ROS accumulation (Liu D. S. et al., 2017). Conversely, it has been widely demonstrated that wtp53 has important role in regulating ROS levels and therefore, in determining the stress response. One mechanism is through the regulation of TIGAR (TP53-induced glycolysis and apoptosis regulator), a transcriptional target of wtp53. TIGAR shares sequence similarities with the bisphosphatase domain (FBPase-2) of the bifunctional enzyme PFK-2/FBPase-2 (6-phosphofructo-2kinase/fructose-2,6-bisphosphatase). These well-known functions lead to glycolysis blockage and favor the production of NADPH through pentose phosphate. This mechanism represents an important mechanism for wtp53 to favor antioxidant capacities, promoting ROS scavenging (Bensaad et al., 2006) Thus, it is not surprising that presence of mutp53 drives an imbalance between glycolysis and oxidative phosphorylation, as well an increase in oxidative stress. Importantly, TIGAR expression has been reported under conditions where mutant versions of p53 are present. Under these conditions, TIGAR plays a key role in protecting cancer cells from oxidative stress generated by sustained proliferation (Cheung et al., 2013). This evidence supports a dynamism between the functions of p53 to adapt to survival under stress conditions.

Recently, it has been shown that cancer cells can adapt to stress conditions. Availability of glutamine in the tumor microenvironment allows cancer cells with mutp53 to generate adaptive mechanisms to avoid apoptosis. However, although glutamine constitutes an important energy fuel for proliferation, cancer cells with mutp53 (R288, R280) can adapt to stress conditions, even in the absence of glutamine. In accordance with this, it was shown that mutp53 can reestablish canonical p53 transcriptional activity over a particular set of genes, such as GLS1, CDKN1A, GAGG45A, and TIGAR, favoring new adaptive mechanisms for stress conditions (Tran et al., 2017; Ishak Gabra et al., 2018).

### Immune System Regulation

**Figure 5:**
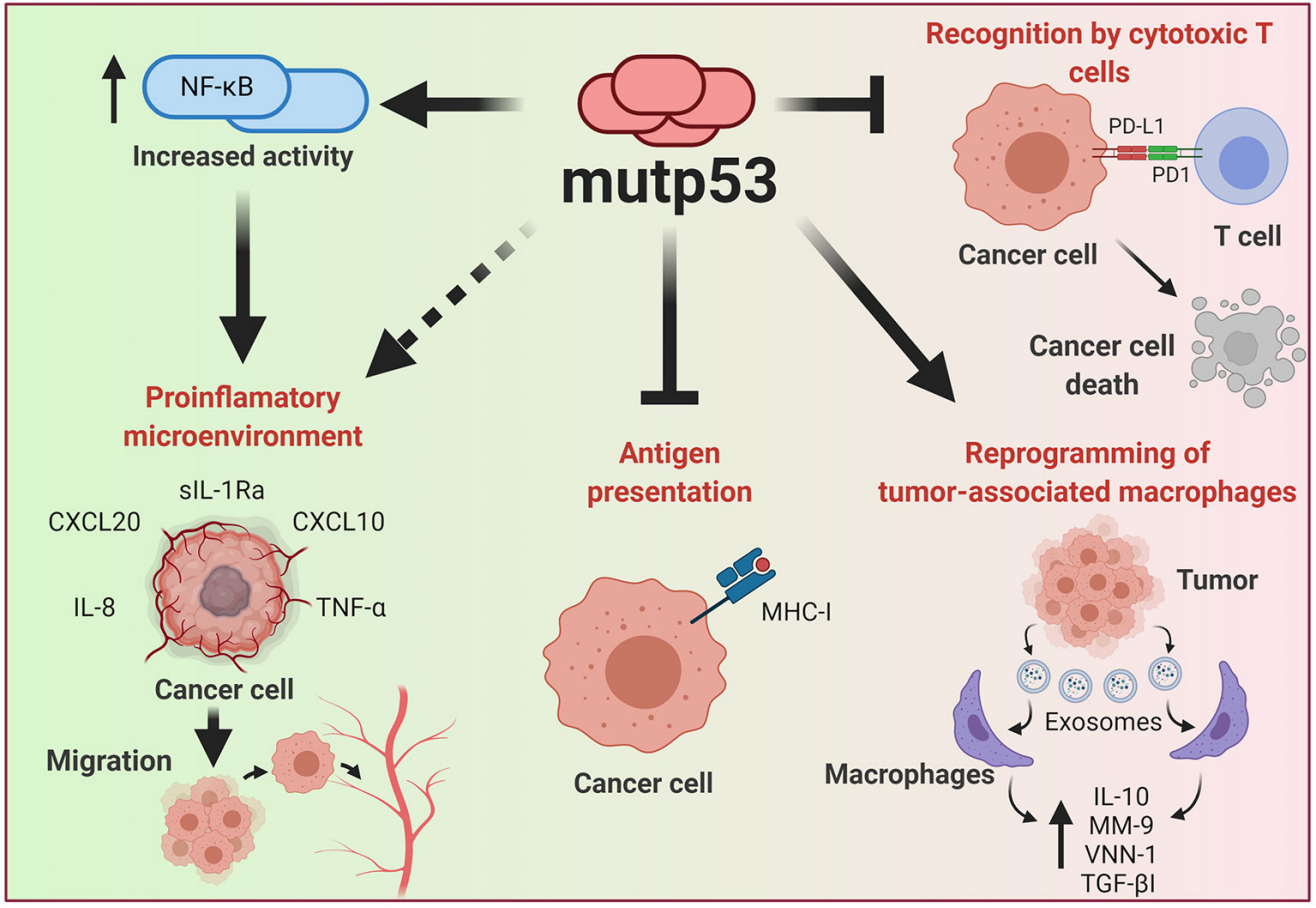
Effect of mutp53 over the immune system. Mutp53 supports a pro-inflammatory microenvironment through the release of siL-1RA, CXCL20, CXXCL10, IL-8, or TNF-α, mainly by increasing the transcriptional activity of NF-kB. Additionally, the presence of mutp53 decreases MHC-I expression, avoiding recognition by T cells. Tumor cells can liberate exosomes that act over neighboring macrophages and improve IL-10, MM-9, VNN-1, and TGF-βI release, thus creating a microenvironment that improves cancer progression. Moreover, mutp53 can increase PD-L1, constituting an important mechanism for avoiding the oncolytic activity of T cells.

Genetic alterations of cancer cells induced by the malignant transformation process has an important effect in the ability to be recognized by the immune system. During recent years, p53 has emerged as one of the major regulators of cancer-immune system interactions (Blagih et al., 2020). The dynamic and bidirectional relationship between tumor cells and the microenvironment has been evidenced to be decisive for the establishment and progression of tumors (Wang et al., 2017). One of the main microenvironment components that allows the development of tumor growth is the immune system; this relationship was already being contemplated in the nineteenth century as a predisposing factor for cancer disease (Gonzalez et al., 2018). It is well accepted that under normal conditions, the immune system seeks to eliminate cells with aberrant characteristics, however, modifications in the functions of neoplastic cells not only prevent elimination, but even benefit from the inflammatory functions of the immune system (Figure 5).

Tumor progression is generally associated with immune system evasion, and loss-of-canonical function of wtp53 stands as a crucial point for the generation of an inflammatory microenvironment that not only limits the immune system response but indeed, benefits cancer cells (Blagih et al., 2020).

As previously discussed, wtp53 acts as a repressor of pro-inflammatory activity through the inhibition of NF-kB, which in turn is also able to inhibit wtp53 activity, thus promoting cell survival and proliferation. This repressive function of wtp53 over NF-kB is impaired by the presence of p53 mutants, acting in an opposite manner, due to the stimulatory effect of mutp53 on NF-kB activity after exposure to TNF-α (Webster and Perkins, 1999; Weisz et al., 2007). The pro-inflammatory and pro-tumorigenic effect of TNF-α, orchestrated by mutp53, results from the interaction between mutp53 and NF-kB. Interestingly, when both factors are bound on promoter regions of cancer-related genes, such as MMP9 and CCL2, they favor an active chromatin, improving transcriptional activity (Cooks et al., 2013; Rahnamoun et al., 2017). The interaction between mut53 and NF-kB is persistent over time, generating inflammation-driven colon cancer (Cooks et al., 2013; Uehara and Tanaka, 2018).

Thus, it is evident that mutp53 tumor cells have greater tumorigenic and migratory capabilities after a pro-inflammatory stimulus, as well as an increase in pro-inflammatory cytokine expression. In addition to TNF-α, IL-8 is also increased in mutp53 cancer cell lines. It is known that IL-8 is a pro-inflammatory cytokine that shows a high capacity for chemotaxis toward neutrophils and whose expression is found to be dependent on NF-kB (Hidaka et al., 2005; David et al., 2016). Consequences of IL-8 over-expression include an increase in tumorigenic properties, the EMT process, as well as improving stemness of tumor cells (Long et al., 2016). Additionally, it has been observed that tumor cells carrying the endogenous R273H mutp53 suppress the activity of sIL-1Ra, an antagonist of the IL-1R (interleukin 1 receptor), through an interaction between mutp53 and the transcription factor MAFF (MAF bZIP Transcription factor F), inhibiting its activity. In this manner, the interaction of the R273H mutant with the MAFF transcription factor, prevents the suppressive action of sIL-Ra on IL-1R, amplifying the pro-inflammatory and tumorigenic activities of IL-1 in colon and breast cancer cell lines (Kannan et al., 2012; Ubertini et al., 2015).

Among the pro-inflammatory cytokines expressed in tumor cells with mutp53 are those which recruit leukocytes, such as macrophages, neutrophils, dendritic cells, and lymphocytes. For example, overexpression of CXCL10, CX3CL1, and LTB chemokines in breast cancer, generates chemotaxis of T lymphocytes, cytotoxic T lymphocytes, as well as NK (Natural Killer) cells, through a mechanism dependent on DAB2IP protein inhibition, thus promoting pro-inflammatory and cell migration activities (Di Minin et al., 2014). This is derived from the DAB2IP repressive activity on pro-inflammatory signaling pathways such as NF-kB, and as previously mentioned, on tumorigenic pathways such as the PI3K/AKT cell signaling pathway. Therefore, the inhibition of DAB2IP through its protein-protein interaction with mutp53 (R176H, R280K), promotes the activation of these pathways (Bellazzo et al., 2017; Valentino et al., 2017). Additionally, in breast cancer cell lines, overexpression of the CXCR4 receptor (whose ligand is the CXCL12 chemokine) has been found in mutp53 cells, improving their migratory capabilities (Mehta et al., 2007).

It has recently been found that this GOF for cytokine release can be accomplished through exosome-mediated mechanisms. Under co-culture conditions of colon cancer cell lines (expressing endogenous mutp53) with M0 and M2 macrophages, it has been observed that the macrophages showed an increase in the release of IL-10, MM-9 (metallopeptidase matrix 9), VNN-1 (non-inflammatory vascular molecule 1), and TGF-βI, due to the action of miR-1246-containing exosomes secreted by tumor cells. This was corroborated in colon tissue samples from patients with mutp53 (Cooks et al., 2018). This anti-inflammatory microenvironment causes a failure in tumor cell elimination by the immune system and, additionally generates activities that favor cellular metastasis due to the destruction of the extracellular matrix and the formation of new blood vessels.

This establishes that mutp53, not only generates a pro-inflammatory environment but also anti-inflammatory ones. Other studies show an over-expression of immunological checkpoints that facilitate immune system evasion by mutp53 tumor cells. In breast cancer patients, over-expression of molecules associated with anti-inflammatory environments such as CTL4, PD-L1, PD-L2, PD-1, LAG2, BTLA, and TIGIT was confirmed in tumors with mutp53, being associated with the prognosis of the disease (Liu et al., 2019).

Similarly, over-expression of the transmembrane protein PD-L1 (Programmed Death-Ligand 1) has been found in mutp53 lung cancer and melanoma cells (Cortez et al., 2016; Thiem et al., 2019). Its main function is the suppression of the pro-inflammatory activity of T cells after the recognition of their specific antigen by interaction of TCR (T cell receptor) with MHC (major histocompatibility complex), being a regulatory mechanism of the inflammatory response (Akinleye and Rasool, 2019). Additionally, the activation of the JAK-STAT pathway by INF-γ receptors generates PD-L1 overexpression (Akinleye and Rasool, 2019). However, in neoplastic cells, mutp53 has been shown to generate low levels of miR-34a, which enables PD-L1 overexpression (Cortez et al., 2016). Additionally, in melanoma, the presence of mutp53 also leads to PD-L1 overexpression and a lower activity of cytotoxic T-cells over tumor cells (Thiem et al., 2019).

Conversely, the positive regulation of wtp53 over MHC-I establishes a relationship between p53 and oncolytic activity by T cells. Taking into account that mutp53 cells show low levels of MHC-I, this could represent an important barrier for T cell recognition. Recently, it has been proposed that low doses of TNF (Tumor Necrosis Factor) can rescue the expression of MHC, making mutp53 cancer cells more sensitive to immunological therapy (Garancher et al., 2020).

This dual role of mutant p53 versions to induce both pro-inflammatory and anti-inflammatory environments becomes a challenge for the immunological eradication of cancer. It is possible that this process is related to the different tumorigenic stages of cancer, and therefore with different microenvironment requirements for tumor progression or favoring certain cellular subsets (Gonzalez et al., 2018).

### Conferring Stemness

It is now accepted that the majority of tumors exhibit a hierarchy of cells within the tumor, where stem-like cells are positioned at the top and are referred to as CSC (Cancer Stem Cells). Under physiological conditions, tissues are subject to constant renewal, and decisions between self-renewal of tissue stem-cells or cell differentiation are associated with wtp53 activity (Solozobova, 2011).

The advancement of genetically modified models provides information about the relevance of the relationship between p53 function and maintenance of the stem cell pool that might provide precursors for tumor initiation. For instance, transgenic mice harboring mutp53 developed malignant glioma, mainly detecting cells in the corpus callosum and olfactory bulb, both migratory destinations for stem cells residing in the subventricular zone. This suggests that neural stem cells or progenitors are mediating gliomagenesis caused by mutp53 (Wang et al., 2009).

Another example was observed in hematopoietic stem cells, where the R172H mutp53 promoted greater ability to self-renew *in vitro* and *in vivo* compared to wtp53 loss, showing that FOXH1, a regulator of stem cell factor receptor c-Kit and SCA-1 (Stem Cell Antigen 1), was necessary for this phenotype in cells expressing mutp53 (Loizou et al., 2019).

Following this notion, mice harboring the R248Q mutp53 favor tumor development, compared to mice with other mutations, such as G245S, since R248Q alters the stem cell compartments, by improving survival and self-renewal of hematopoietic and mesenchymal stem cells, putative primary malignant cells. This explains the similarity with Li-Fraumeni patients, a familial cancer predisposition, in which the R248Q mutp53 increases tumor initiation compared to other mutants, possibly because the R248Q mutp53 is able to co-aggregate into higher-order structures with other tumor-suppressor transcription factors (Xu et al., 2011; Hanel et al., 2013).

Some types of cancer are originated through age-related mutations. This can be evidenced in C57BL/6, a type of old mice vulnerable to developing fibrosarcoma, where it was demonstrated that mesenchymal stem cells isolated and cultured *in vitro* were spontaneously transformed. The acquisition of tumorigenic potential was accompanied by the expression of stemness factors such as Klf4, Oct4, Sox2, c-Myc, as well as by the expression of mutp53 (Li et al., 2007).

The self-renewal capacity of undifferentiated populations requires a balance between “open” and “closed” state of the chromatin. During the stemness of embryonic cells, a bivalent state has been identified that reflects posttranslational modifications of histones that can generate a transcriptionally inactive state. PRC1 and PRC2 (Polycomb Repressive Complex 1 and 2) act in an orchestrated way to keep this repressive state, defining specific lineages. However, in the case of cancer, these mechanisms can regulate oncogenic functions through silencing of tumor suppressor genes (Laugesen et al., 2016). Interestingly, the presence of wtp53 seems to be determinant in controlling these epigenetic modifications.

Recently, it has been found that mutp53 triggers self-renewal of hematopoietic stem cells by increasing levels of H3K27me3 and therefore promoting a repressive chromatin state. In this study, three mutp53 versions (R248W, R273H and R175H) showed increased association with EZH2 (Enhancer of Zeste Homolog 2), which is part of PCR2 (Polycomb Repressive Complex-2), compared with wtp53, improving EZH2 binding to chromatin. Nonetheless, mutp53 promoted the presence of H3K27me3 rather than altering genomic distribution (Chen et al., 2019). Moreover, mutp53 also indirectly upregulates EZH2 by attenuating miR-26a, a negative regulator of EZH2, supporting another mechanism in the regulation of EZH2 activity (Jiang et al., 2015).

One of the main challenges in the study of CSC has been the development of appropriate tools that allow distinguishing them from the rest of the cancer cells. Surface protein markers have allowed addressing this problem, predominantly employing CD44, LGR5, and CD133 (Barker et al., 2007; Keysar and Jimeno, 2010; Alvarado-Ortiz et al., 2019). It was shown that wtp53 inhibits CD44 expression in breast cancer cells, but R248H mutp53 increased CD44^+^ cells in colorectal cancer (Zeilstra et al., 2013; Solomon et al., 2018).

Solomon and collaborators showed the relationship between p53 functionality and CSC properties in colorectal cancer. They showed that in the RKO cell line, which endogenously expresses wtp53 but was transfected with R248H mutp53, there was an increase in the number of LGR5^+^ and CD44^+^ cells. Conversely, knockdown of endogenous mutp53 in SW480 cells diminished CD44^+^ cells (Solomon et al., 2018).

**Figure 6:**
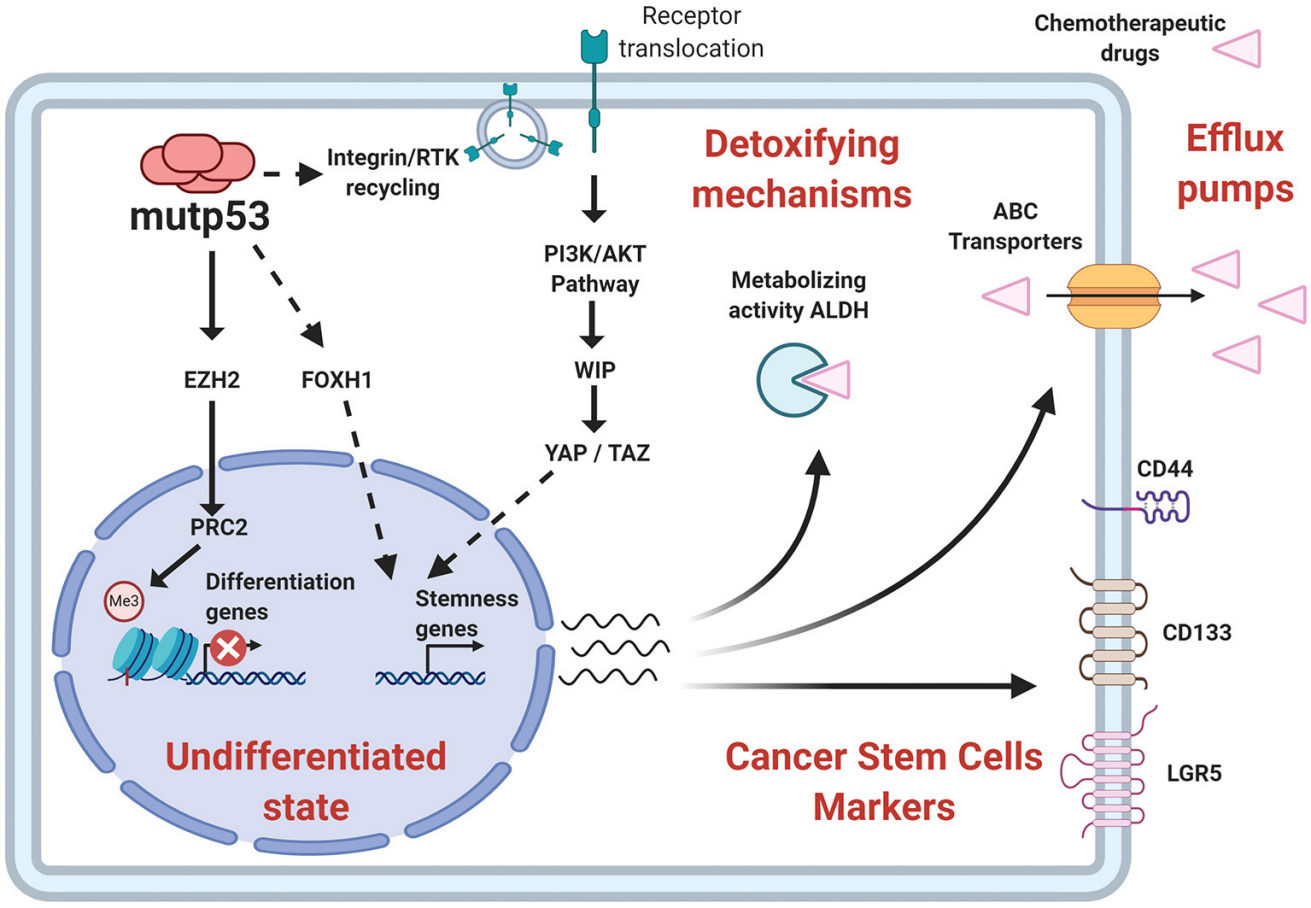
Relationship of stemness properties with mutp53. Mechanisms related to self-renewal pathways favored by mutp53 include an increased EZH2 (subunit of PCR2 complex) activity and improvement of epigenetic modifications associated with a repressive state of chromatin. Other pathways include YAP/TAZ activity, as well as nuclear effects of FOXH1. Additionally, mutp53 increases CSC markers, such as CD44, CD133, LGR5, and the enzymatic activity of ALDH, contributing to stemness and pharmacological resistance.

In addition to surface proteins, CSC can also be identified through high enzymatic activity of proteins such as ALDH (Aldehyde Dehydrogenase) (ALDH^HIGH^ cells) (Toledo-Guzmán et al., 2019). The ALDH^HIGH^ population was augmented in cells that overexpress mutp53, while it was reduced by mutp53 knockdown (Solomon et al., 2018; Zhao et al., 2019). Furthermore, ALDH levels were upregulated in colorectal tumor samples expressing p53 missense mutations and clinically associated to higher aggressiveness. This poor prognosis seems to be linked to CSC-related capabilities, such as higher tumorigenic potential and chemo-resistance, which agrees with the proposal that mutp53 favors chemo-resistance and its absence leads to chemo-sensitivity (Figure 6).

Interestingly, mutp53 binds to promoter sequences of ALDH1A1, CD44, and LGR5. ALDH1A1 being implicated in mutp53-mediated chemo-resistance (Chen et al., 2016; Solomon et al., 2018). ALDH1A1 belongs to the ALDH family of enzymes and is capable of metabolizing, not only endogenous substrates but also inactivates some drugs used in chemotherapy, especially aldophosphamides. Remarkably, Gui et al. (2020) found that while the presence of wtp53 preferentially associates with a dominant ALDH isoform in tumors from HNSCC (Head and Neck Squamous Cell Carcinomas), mutp53 displayed a different diversity of ALDH isoforms, thus severely influencing chemoresistance associated to ALDH.

Chemo-resistance involves capabilities such as efflux of exogenous agents. Therefore, artificial dyes are used to identify these cells *in vitro* and CSC are then referred to as SP (Side Population). Analysis of SP in colorectal cancer-derived cell lines show that DLD-1 cells expressing mutp53 and Caco-2 (p53 null) cells showed a SP, while in HCT116 cells harboring wtp53, it was hardly detected. This is in agreement with the evidence that wtp53 inhibits MDR genes. Therefore, alterations in the p53 gene would impact the drug efflux capacity of the cells (Allen et al., 2009).

Functionally, CSC also are capable of serially forming spheres *in vitro* and display higher tumorigenic potential under *in vivo* conditions. An example was confirmed by Zhao et al. (2019) where overexpression of R273H mutp53 in a p53-null cell line showed elevated sphere formation and an increased expression of Sox2 and Nanog, favoring greater tumor initiating capacity. Furthermore, normal astrocytes expressing mutp53 were able to form spheres, emphasizing the potential of mutp53 in non-stem cells to trigger a CSC-like state (Escoll et al., 2017).

Supporting evidence for molecular mechanisms that allow self-renewal in the presence of mutp53 are scarce. The canonical Wnt pathway represents the most likely candidate, since it is commonly associated with self-renewal of stem cells, and its activity seems to be dependent on p53 status (Nusse and Clevers, 2017). The β-catenin protein is the transcriptional cofactor involved in Wnt signal transduction, and is modified post-translationally to regulate its functions.

It is well known that wtp53 acts negatively in regulating the canonical Wnt pathway (Kim et al., 2011). It is noteworthy that mutp53 increases β-catenin levels (Cagatay and Ozturk, 2002), possibly through Siah1 (Seven in absentia homolog 1) regulation, a target of wtp53 that participates in β-catenin degradation (Fiucci et al., 2004; Xie et al., 2009). Another mechanism related to β-catenin levels is mediated by mir-34a, a p53 transcriptional target, which downregulates β-catenin mRNA. In this regard, it is striking that mir-34a targets additional components of the Wnt pathway, decreasing β-catenin-dependent transcription, including stemness-related genes (Kim et al., 2011).

Therefore, the inhibitory role of wtp53 over the Wnt pathway is consistent with an increase in transcription mediated by β-catenin in the presence of mutp53 (Cagatay and Ozturk, 2000). Furthermore, c-Myc and Oct4 are upregulated in cells that express mutp53, which could be explained by upregulation of β-catenin activity, but additional experimental data are required to demonstrate this signaling axis (Hosain et al., 2016).

Some *in vivo* models have been employed that overactivate Wnt signaling to generate tumors in mice. Wnt-1 transgenic mice develop mammary cancer, but when the mice are additionally modified with a mutant version of p53 (R175H), a higher number of tumors in many mammary glands are observed. Additionally, there is an increase in the pool of mammary epithelial stem cells, which is related to tumorigenic potential (Lu et al., 2013).

Similarly, synergistic oncogenic properties of mutp53 have been shown in two additional mouse models of Wnt-driven gastrointestinal cancer. In these studies, there are exogenous conditions related to microenvironment, such as gut microbiome, that determine the functions of mutp53 in two different anatomical sites, intestine and colon. The microorganisms of the intestinal tract and colon are interacting with the host cells maintaining homeostasis. Recently, it was evidenced that a microbiome imbalance can promote tumorigenesis, and its anatomical localization has proven to be a key factor to promote the oncogenic effect of mutp53. In this study, mutp53 GOF was dependent on gallic acid, a metabolite that simulates the effects of the microbiome in the gut. This became evident because mutp53 strengthened tumor occurrence in distal sites, like colon, characterized by gallic acid enrichment, while mutp53 diminished tumor progression into proximal sites, where gallic acid is scarce. In the proximal site, the tumor-suppressive activity of mutp53 was independent of canonical p53 transcription and more closely associated with suppression of the Wnt pathway. TCF4, the transcriptional factor mediating β-catenin activity, was shown to be decoupled from chromatin and the H3K4me3 active transcription epigenetic marker was dropped from Wnt response targets. Noticeably, this mechanism of Wnt signaling inhibition was not observed in crypts, in the presence of gallic acid, diminishing the protective task of mutp53 against tumorigenesis and conversely, promoting tumor development, as reflected by canonical Wnt targets, such as CD44, c-myc, or Axin2. The overall data postulate a dual role for mutp53, in which the exogenous components and the effects of the microbiome, though gallic acid, might decide the transition from tumor-suppressive to oncogenic function, or vice versa (Kadosh et al., 2020).

The YAP/TAZ complex (Yes-Associated Protein/Transcriptional Co-Activator with PDZ-binding motif), is a key component of mechanical stress. YAP/TAZ complex is important for self-renewal and tumorigenic capacity (Cordenonsi et al., 2011). Escoll et al. (2017) described that mutp53 stimulates YAP/TAZ stability through phosphorylation of WIP (WASP-Interacting Protein) by AKT2, in glial and breast cancer cells. In this context, mutp53 upregulated CSC marker (CD133 and CD44) expression, sphere formation and tumor capability. Interestingly, in this study mutp53 requires membrane-associated components to transmit the signal to WIP, similar to oncogenic pathway related GOF. This evidence indicates a mechanism by which mutp53 can also regulate CSC self-renewal.

The loss of p53, as well as GOF attributed to mutp53, seems to be necessary for the presence of tumor-initiating properties, as recently described in intestinal cancer. The loss of p53 function in addition to mutp53, promotes a greater ability to form organoids and increases tumorigenic capabilities, since the presence of one functional allele of *TP53* with mutp53 does not increase these characteristics. Particularly, this genotype shows elements related to stemness and inflammatory pathways. This evidence is not trivial, since clonal expansion, survival and metastatic effects are attributed to mutp53 in both alleles (Nakayama et al., 2020).

## Gain-of-Function and Chemoresistance

### Mechanisms of Chemoresistance

**Figure 7:**
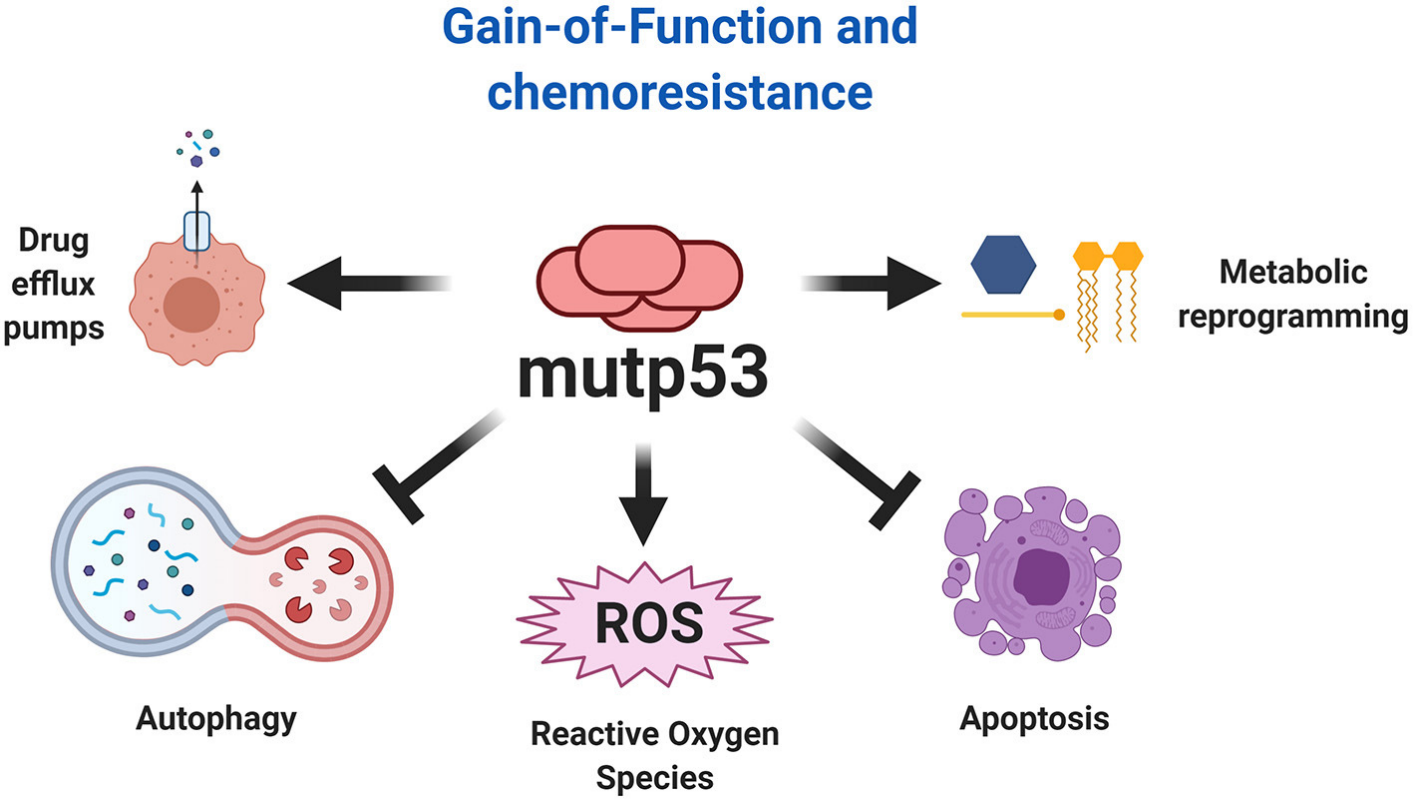
Chemoresistance explained by mutp53. The gain-of-function of mutp53 has been broadly implicated in various mechanism of chemoresistance, including resistance to apoptosis, autophagy inhibition, metabolic reprogramming, and increased expression of drug efflux pumps. Additionally, the effect of mutp53 and the downstream effects of gain-of-function favors autophagy inhibition and ROS production, being an important mechanism that explains the overactivation of oncogenic pathways.

Resistance to chemotherapy is a major cause of cancer-associated death that can occur due to several factors, including enhanced drug efflux and metabolic reprograming (Holohan et al., 2013). Evidence indicates that GOF activities promote tumor progression and can drive resistance to a variety of anticancer drugs (Figure 7). In fact, many studies show that mutp53 is associated with increased expression of MDR1 (multidrug resistance gene 1), an important drug efflux pump (Sampath et al., 2001). It was discovered that mutp53 knockdown reduces cell proliferation and resistance to cisplatin, adriamycin and etoposide in several cancer cells lines (Bossi et al., 2006). In humans, colorectal carcinomas harbor frequent mutation of *TP53* that are associated with resistance to tyrosine kinase inhibitors (Wang B. et al., 2013).

In a prospective study including patients with locally advanced breast cancer, it was observed that those with mutp53 status treated with doxorubicin and a combination of 5-fluorouracil and mitomycin, showed a reduced recurrence-free and overall survival in comparison to patients with wtp53 tumors (Eikesdal et al., 2014). Moreover, it has been reported that the K351N mutp53 may be associated with induction of platinum resistance in patients with advanced epithelial ovarian cancer (Zhang et al., 2014).

Although impact of mutp53 on drug resistance has been widely reported, it is important to understand the mechanisms through which such resistance is achieved. Some p53 mutants provide enhanced resistance to apoptosis induced by a variety of chemotherapeutic drugs (Wang Q. et al., 2013). Interestingly, it was shown in a squamous cell carcinoma model, that overexpression of the R273H mutant is associated with doxorubicin and methotrexate resistance through the inhibition of apoptosis by procaspase-3 downregulation (Wong et al., 2007). In accordance with this, Donzelli and coworkers (2012) showed that mutp53 also confers chemoresistance to doxorubicin, cisplatin and 5-fluorouracil by procaspase-3 downregulation. Specifically, they demonstrated that the R175H mutp53 is able to induce miR-128-2 expression, which in turn upregulates p21, promoting its accumulation in the cytoplasm favoring cellular survival (Donzelli et al., 2012).

Since DNA damage is one of the central mechanisms of current chemotherapeutic drugs, up-regulation of DNA repair function promotes resistance to these agents. Interestingly, a recent study showed that the mechanism of resistance to adriamycin in breast cancer with mutp53 was through downregulation of miR-30c and translesion synthesis DNA polymerase, REV1, indicating that mutp53 favors the DNA damage repair pathway (Lin et al., 2019). Moreover, Yan et al. (2018) demonstrated that inhibition of UDG (Uracil DNA Glycosylase) selectively sensitized mutp53 cancer cells to 5-FdU (Floxuridine), but did not alter the response in wtp53 cancer cells. Since UDGs are DNA repair enzymes, that also recognize 5-FdU to initiate the base excision repair pathway, these enzymes are important for the effect mediated by 5-FdU. Thus, UDG depletion restores sensitivity and has chemotherapeutic potential in the context of cancer with mutp53. Previous studies have indicated that mutp53 decreases chemosensitivity of glioblastoma to temozolomide by increasing the expression of MGMT (O6-methylguanine DNA-methyltransferase), this enzyme is involved in repair of DNA damage caused by temozolomide, thereby contributing to drug resistance (Wang et al., 2014).

In other studies, it has been shown that several p53 mutants, such as R273H, C176S, and R248W, promote gemcitabine resistance in pancreatic adenocarcinoma cells lines. Specifically, gemcitabine stabilizes mutp53 and promotes its phosphorylation at Ser15, mutp53 then induces CDK1 (Cyclin Dependent Kinase 1) and CCNB1 (Cyclin B1) gene expression promoting a hyperproliferation effect and chemoresistance (Fiorini et al., 2015).

Recently, an effect of mutp53 over EFNB2 (Ephrin-B2), which is a receptor tyrosine kinase that regulates invasion, migration, angiogenesis and tumor resistance has been reported (Zhu et al., 2020). Moreover, it was reported that mutp53 increases EFNB2 expression in colorectal carcinoma cell lines, when the cells were treated with 5-FU (Alam et al., 2016). EFNB2 induces 5-FU resistance through the upregulation of the ABCG2 (ATP-binding Cassette Sub-family G-2) multi-drug resistance efflux transporter, mediated by the activation of the c-Jun/JNK signaling pathway (Alam et al., 2016). Recently, it was observed that mutp53 upregulates the receptor tyrosine kinase AXL in colon and breast cancer models, and also contributes to the EMT process, impairing the response to therapy. Interestingly, expression of AXL confers an invasive potential to mutp53 cells after exposure to chemotherapy, compared to cells where AXL was silenced. In support of this, mutp53 increases AXL expression at mRNA and protein levels (Zhu et al., 2019).

The survival of cancer cells can be modulated by Ca^2+^ dynamism, which represents an important influence on chemoresistance. The excessive transfer of Ca^2+^ to the mitochondria drives pro-apoptotic responses. Interfering with this process improves mechanisms related with cellular survival, including autophagy. The role of Ca^2+^ depends on p53 functionality. Wtp53 increases Ca^2+^ transfer from endoplasmic reticulum to mitochondria, promoting apoptosis under stress conditions (Bittremieux and Bultynck, 2015). In the absence of p53 and the presence of mutp53 (R175H, R273H), cancer cells fail to transfer Ca^2+^ to the mitochondria, favoring chemoresistance to stressor treatments (Giorgi et al., 2015). This mechanism can be useful in therapy to control the effect of pharmacological treatments.

It was reported that p53 mutants establish amyloid-like aggregates that contribute to cancer progression and tumor resistance (Levy et al., 2011; Yang-Hartwich et al., 2015). These aggregates sequester native p53 protein into an inactive conformation lacking pro-apoptotic function, leading to platinum resistance in high-grade serous ovarian carcinoma cells (Yang-Hartwich et al., 2015). More importantly, mutations in p53 may shift the conformation distribution favoring the generation of aggregates. For instance, a recent study found the presence of amyloid-like mutant p53 in brain tumor cells showing chemoresistance to temozolomide (Pedrote et al., 2020). Interestingly, Zhang et al. (2017) identified ERP29 (endoplasmic reticulum protein 29) as a key mediator of chemoresistance by the aggregation prone R282W mutp53 and suggest that targeting ERP29 may sensitize cancer cells to cisplatin treatment. They show that R282W mutp53 upregulates ERP29 at the mRNA and protein expression levels. Conversely, using ReACp53, a peptide inhibitor for p53 aggregation, they observed a decrease in ERP29 and ultimately the reversion of chemoresistance.

Recently, it was shown that the R273H mutant induces 5-FU resistance in colorectal cancer through the downregulation of the proapoptotic protein PUMA. Employing ChIP assays, they show that mutp53 could not bind to the PUMA promoter, which impairs its transcription. PUMA is a critical upstream activator of the proapoptotic protein BAX, thus mutp53 decreases BAX activity and hence the apoptotic process (Huang et al., 2019). The fact is that several reports show that PUMA induction by chemotherapeutic agents and adenoviral delivery assays, suppresses tumor growth and sensitizes to chemotherapy through induction of apoptosis in head and neck cancer (Sun et al., 2007).

Nuclear effects of mutp53 can increase chemoresistance, since mutp53 can act with the YAP/β-arr1 complex to improve its transcriptional program over the TEAD transcription factor. This effect seems to be explained by the ET-1R (Endothelin-1 Receptor) signaling pathway, a mechanism related to the increase in YAP activity. This process promotes resistance to cisplatin, as was recently described in ovarian cancer. The use of ET-1R antagonists, such as macitentan, affects the nuclear YAP/mutp53/β-arr1 complex, making cancer cells more sensitive to cisplatin and representing an important axis that can be disrupted in patients with this type of cancer. This is not just another mechanism, since it offers an explanation of where mutp53 pathways interconnect, for improve chemoresistance (Tocci et al., 2019).

### Autophagy Inhibition by Mutant p53

The role of autophagy in cancer depends of the types of oncogenes observed, which offers interesting views, from a metabolic perspective, related to chemoresistance (White, 2016). The GOF of mutp53 promotes autophagosome inhibition. However this process is much more complex when we try to understand the metabolic consequences related to autophagy inhibition, since in different types of cancer “autophagy addiction” is necessary for tumor cell survival (Santana-Codina et al., 2017).

Autophagy is a catabolic process where macromolecules and organelles are degraded into lysosomes, providing energetic fuels under stress conditions (Santana-Codina et al., 2017). Autophagy is determinant for the progression of some types of cancer. For example, continuous proliferation is a characteristic of Ras-driven cancer types, in which intracellular autophagy markers are increased, providing biosynthetic precursors, intermediaries of TCA (tricarboxylic acid) cycle, and nucleotides critical for growth and survival during stress conditions, such as starvation (Guo et al., 2011, 2016; Yang et al., 2011). In this sense, pancreatic ductal adenocarcinoma tissues and cell lines reveal increased autophagosomal markers, which is determinant for malignant progression, since autophagy inhibition compromises growth and tumorigenicity (Yang et al., 2011).

There is a relationship between autophagy and p53 functions, since wtp53 induces autophagy and conversely, autophagy inhibits wtp53 activity. Moreover, wtp53 functions favor autophagy through transcriptional response, this process ensures cellular homeostasis under stress damage. In the case of cancer, wtp53 can limit oncogenic transformation and autophagy driven by K-Ras (White, 2016). Thus, oncogenes and tumors suppressor genes can influence the role of autophagy in cancer.

The increases in autophagy flux represents an important element in the development and progression of Ras-driven cancer types. However, this is not the case when there is a loss of p53 function. Interestingly, in cell line and mice models that harbor mutated K-Ras, it has been reported that autophagy is not active under conditions where p53 is not functional, resulting in an important decrease of autophagy protein markers as well as autophagosomes. Interestingly, Rosenfeldt et al. (2013) observed that autophagy inhibition is crucial for pre-cancerous pancreatic intraepithelial neoplasia and favors pancreatic adenocarcinoma, potentiating tumor cell growth, even more than the autophagy induced by K-Ras. This evidence allows us to establish that pharmacological inhibition of autophagy in cancer may be counterproductive. Therefore, anabolic requirements generated by the absence of wtp53 must be considered. In the case of Ras-driven cancer, the requirement for oxidative metabolism through mitochondrial function, as determined by oxygen consumption, along with the ATP and macromolecule biosynthesis requirements, explain the “autophagy addiction,” so these types of cancers are more sensitive to autophagy inhibitors (Guo et al., 2011, 2016; Yang et al., 2011).

In tumor cells with non-functional p53, metabolic requirements seem to be a consequence of the Warburg effect, since the dual effect of a K-Ras mutation and absence of wtp53 increases glucose uptake, releasing lactate as a metabolic precursor to sustain anabolic pathways, while oxygen consumption or synthesis of TCA cycle intermediaries are not affected. Autophagy inhibition combined with the absence of p53 increases the tumorigenic capabilities of K-Ras driven cells, as determined by lower overall survival of mice with pancreatic adenocarcinoma (Rosenfeldt et al., 2013; White, 2016).

The status of p53 is determinant for the correct use of autophagy flux inhibitors as a tool that could compromise the proliferative capacities of cancer cells, since autophagy inhibition can increase premalignant lesions in pancreas due to Ras oncogene and loss of p53, but the capacity to acquire an invasive phenotype is impaired. Yang et al. (2014) argue that autophagy inhibition can be employed independently of TP53 status, reducing the oxygen consumption rate and clonogenicity (Yang et al., 2011, 2014; Rosenfeldt et al., 2013). Similarly, it has been suggested that the reestablishment of canonical functions of wtp53 on tumor cell lines with different TP53 mutants, using reactivator molecules, could sensitize cancer cells to apoptosis. It has been observed that dual administration of these treatments with autophagy flux inhibitors have important effects for reducing the proliferative capability. However, this effect is not observed with the individual use of wtp53 reactivator molecules. This leads to positioning autophagy as a survival process, so that unique treatments based on reestablishing wtp53 seem to be therapeutically unviable (Fiorini et al., 2013).

Determination of mutp53 is critical for elucidating the differential effects observed over autophagy. While mutp53 proteins that localize to the nucleus enhance autophagy, those that localize to the cytoplasm decrease it (Morselli et al., 2008). One of the proposed mechanisms explaining the downregulation of autophagy related to GOF is through autophagosome protein markers, like Atg12 and Beclin1, among others. The expression of autophagosomal markers is NF-κB dependent, however, mutp53 binds to p50 (NF-κB subunit), promoting repression of Atg12 expression. In agreement with other reports, mutp53 downregulates AMPK activation, which is reflected by an increase in mTORC1 activity, a suppressor of the autophagy process. Taken together, these reports sustain a molecular basis by which autophagy is reduced in the presence of mutp53. Interestingly, different cancer cell lines with mutp53 are more sensitive to inhibition of mTORC1 (Zhou et al., 2014; Cordani et al., 2016).

The inhibition of mTORC1 could generate an increase in autophagic flux and consequently contribute to pharmacological resistance. For example, it was recently shown that overexpression of miR-338-3p confers 5-FU (5-fluorouracil) resistance in colorectal cancer cells with p53 mutant status by targeting the mTORC1 pathway (Han et al., 2017). Consequently, mTOR impairment resulted in an increase in autophagy. They showed that the mechanism influencing 5-FU sensitivity was due to competition between autophagy and apoptosis. In this case, autophagy could be playing a key role in protecting cancer cells from stress-induced damage caused by 5-FU (Han et al., 2017).

## Therapeutic Perspectives

Mutp53 GOF is observed in most human cancers and generates a dependence for tumor maintenance by several mechanisms shown in Table 1. The inhibition of mutp53 represents an effective strategy for therapy. Currently, there are different therapeutic strategies focused on targeting mutp53. We have decided to classify them into three categories: restoring wtp53 functions, disrupting REDOX balance, and targeting mutp53 for degradation (Mantovani et al., 2019; Zhang C. et al., 2020). Based on this, there are several clinical trials registered (ClinicalTrials.gov), and some of them use these strategies in combination with common chemotherapeutic treatments to prevent resistance to the current therapy.

**Table 1:** Mutant p53 gain-of-function.

**Gain-of-function**	**Molecular mechanism**	**Mutant version**	**Type of cancer**	**References**
Proliferation	Increasing receptors translocation through the RCP complex	R175H R273H	Breast cancer	Muller et al., 2009
	Increasing the PI3K/AKT axis through inhibition of DAB2IP	R280K R175H	Breast and prostate cancer	Valentino et al., 2017
	Increasing the active state of K-Ras	R175H	Pancreatic cancer	Escobar-Hoyos et al., 2020
Migration and metastasis	Interacting with p63 and downregulating its anti-metastatic activities	R175H	Breast cancer	Gaiddon et al., 2001; Adorno et al., 2009
	Increasing Rac1 activity	R175H R248W R273H	Colorectal cancer	Yue et al., 2017
	Interacting with HIF1α and favoring secretome activity and metastatic capabilities	R175H R273H R280K	Breast cancer	Capaci et al., 2020
Metabolic reprogramming	Increasing activity of RhoA/ROCK axis and translocation of glucose transporters to membrane	R175H R248Q R273H	Lung and breast cancer cells	Zhang et al., 2013
	Interacting with SREBP and increasing the MVA pathway	R273H	Breast cancer	Freed-Pastor et al., 2012
	Inhibiting AMPK activity and increasing anabolic pathways	R175H G245C R282W	Head and neck squamous cell carcinoma	Zhou et al., 2014
Immune evasion	Increasing inflammation and favoring NF-kB activity	R273H	Colorectal cancer.	Cooks et al., 2013
	Augmenting pro-inflammatory activity in the tumor microenvironment by interaction with MAFF	R273H	Colon and breast cancer cells	Ubertini et al., 2015
	Reprogramming macrophages from M1 to M2	R245 R248 R175 R273 R282	Colorectal cancer	Cooks et al., 2018
Stemness	Increasing CSC surface markers and ALDH enzymatic activity	R175H R273H	Colorectal cancer	Solomon et al., 2018
	Increasing activity of YAP/TAZ pathways and promoting self-renewal of CSC	R175H R273H	Glioblastoma and breast cancer cells	Escoll et al., 2017
	Inducing a repressive state of chromatin through PRC2 activity	R175H R248W R273H	Hematopoietic stem cells	Chen et al., 2019
Chemoresistance	Favoring changes in transcriptional regulation by mutp53/YAP/β-arr1 and promoting cisplatin resistance	R273H	Ovarian cancer	Tocci et al., 2019
	Up-regulating DNA repair pathways	R280K	Breast cancer	Lin et al., 2019
	Downregulating procaspase-3 by increasing miRNA-128-2	R175H	Non-small-cell lung cancer	Donzelli et al., 2012

### To Restore or Not to Restore *Wild Type* p53 as Therapy

Therapeutic strategies for treatment of tumors with mutp53, include reestablishing normal p53 functions. Presence of missense mutations in p53 destabilizes the zinc interaction, resulting in misfolding and loss of a *wild type* tridimensional structure (Joerger and Fersht, 2008). Interestingly, supplemental zinc in culture media has been shown to restore *wild type* structure in some mutp53 cells, as well as its corresponding transcriptional activity (Margalit et al., 2012). Innovatively, it was found that treatments such as Zn-cur (Zinc-curcumin complex) induce a structural change from mutp53 to wtp53, restoring its canonical functions. This type of treatment can even cross the blood-brain barrier, and in the case of the glioblastoma model could be an important treatment strategy (Garufi et al., 2013).

Similarly, another metal ion chelator is COTI-2, which can bind to misfolded mutp53 forms and restore p53 activity as a transcription factor, leading to reactivation of wtp53 target genes such as p21, PUMA, and NOXA. COTI-2, has been tested in preclinical phase studies on different types of cancer (Lindemann et al., 2019). For instance, one clinical trial has used it as monotherapy or combined with cisplatin in lung and colon cancer patients with recurrent malignancies (NCT02433626).

Another proposal is the use of PRIMA-1 and its analog ARP-246, whose mechanisms of action is through the MQ (methylene quinuclidinone) metabolite. Mutp53 can recover its transcriptional activity through the generation of adducts, which increase DNA-binding, through an alkylation process that favors its correct folding. Once inside the cell, PRIMA-1 is degraded to MQ, and interacts with the thiol chemical groups of mutp53, thus promoting recovery of its transcriptional activity (Lambert et al., 2009).

APR-246 is considered to be a promising first-in-class mutp53 targeting drug, since it is more potent and less toxic than PRIMA-1 (Lambert et al., 2009). Currently, there are ten clinical trials in phase I and II for different types of cancer registered at ClinicalTrials.gov (NCT03268382, NCT02098343, NCT00900614, NCT04214860, NCT04383938, NCT03072043, NCT03588078, NCT03745716, NCT03391050, NCT04419389). The combined use of APR-246 and Pegylated Liposomal Doxorubicin Hydrochloride (PLD) has been used on ovarian cancer patients with mutp53 (NCT03268382 and NCT02098343), however the effectiveness is not yet well defined.

Other report supports that acetylation of mutp53 (R158G) through pharmacological agents alters its ability to bind DNA, decreases the oncogenic effect of NF-kB activity, and finally, favors apoptosis, making cancer cells sensitive to DNA-damaging agents. The particular effects of mutant p53 versions can be employed for the use of specific treatments focused on their GOF (Kong et al., 2020).

Reports that focus on reestablishing wtp53 functions have determined that PEITC (Phenethyl isothiocyanate) can favor canonical p53 activity in cell line models expressing endogenous mutp53, by promoting a wtp53-like conformational state. This effect recapitulates the ability to be regulated by ubiquitination and even to reduce the tumorigenic ability observed in xenograft models (Aggarwal et al., 2016, 2019).

Recently, it has been shown that wtp53 improves metabolic adaptation and favors tumor progression by PUMA regulation, a proapoptotic protein overexpressed in hepatocellular carcinoma, and by increasing mitochondrial activity through oxidative phosphorylation (Bensaad et al., 2006; Puzio-Kuter, 2011). However, overexpression of PUMA and wtp53 phosphorylation by IKKβ promotes its binding to MPC (Mitochondrial Pyruvate Carrier), which impairs the transport of pyruvate to the mitochondria. The functional relevance of this mechanism is to drive a preponderantly glycolytic pathway, avoiding oxidative phosphorylation as a major fuel for ATP. This supports the survival process related with wtp53 in this type of model. The effect of wtp53 over PUMA can act as an oncogenic axis, since wtp53 or PUMA knockdowns can preclude, not only this metabolic switch, but also tumorigenic capability, making it challenging to restore wtp53 functions with the purpose of therapy (Kim J. et al., 2019).

New reports reveal that in cancers expressing wtp53, such as melanoma, its overactivation favors a slow-cycling phenotype, a population characterized by low proliferation but higher invasive capabilities that result in pharmacological resistance. Increasing wtp53 induces cellular arrest and senescence, but not a proapoptotic effect, suggesting a dynamism favoring survival. In this case, impairing wtp53 together with the use of MAPK inhibitors could be important to reduce the metastatic capabilities of melanoma cells. This evidence suggests an alternative role for wtp53 that drives chemoresistance (Webster et al., 2020).

Another report sustains that under stress conditions caused by low availability of glutamine, the transcriptional activity of wtp53 and mutp53 can be restored through phosphorylation by IKKβ, regulating genes related with survival of cancer cells, while proapoptotic genes are not expressed (Ishak Gabra et al., 2018). This evidence allows us to establish a solid molecular base by which the reestablishment of wtp53 canonical functions could have counterintuitive effects on metabolic adaptation. This suggests that the use of treatments based on improving wtp53 function is not the only answer, and it is necessary to define the p53 functional status in cancer in order to understand the possible mechanisms that could result in pharmacological resistance.

### Disrupting the Redox Balance

As a consequence of the presence of mutp53, ROS accumulates due to both, loss of wtp53 antioxidant capacities and mutp53 GOF. This includes antioxidant enzyme imbalance, inhibition of autophagy, and the overactivation of oncogenic pathways (Cordani et al., 2020).

It is important to highlight that the hyperproliferation process related to mutp53 involves an increase in ROS. Simultaneously, the presence of mutp53 suppresses antioxidant responses through the repression of Nrf2 activity, as well as glutathione synthesis, both implied in the improvement of antioxidant mechanisms (Liu D. S. et al., 2017). One of the goals of preventive treatment is the upregulation of canonical p53 functions, with the purpose of favoring antioxidant capabilities as a protective tool for responses such as DNA damage. In this manner, in the face of oncogenic damage it would be generating apoptosis or cell cycle arrest (Budanov, 2014). For example, employing the natural phenolic compound curcumin, an antioxidant agent with anti-inflammatory properties, there was an increase in p53 half-life, given by interaction with the NQO1 protein. This mechanism is an important element to improve the decrease in cell viability observed in cell lines with wtp53 (Patiño-Morales et al., 2020).

However, continuous treatment to reestablish the p53 functions could result in pharmacological resistance, since the increase in antioxidant activity in cancer cells would prevent cell death caused by exogenous stress generated by radiotherapy or the oxidant stress of a chemotherapeutical treatment (Conklin, 2004; Trachootham et al., 2009). Moreover, increasing antioxidant ability protects against ROS through wtp53, but may lead to apoptosis resistance. Additionally, p21, a transcriptional target of wtp53 can promote mechanisms related to survival, such as senescence. Therefore, one must be careful when using pharmacological treatments for different types of cancer where the functional state of p53 is not well known. The importance of REDOX balance could be employed to compromise mutp53 function (Vousden and Prives, 2009).

For instance, the hypoxia process favors cell cycle arrest and pharmacological resistance, since it impairs the proliferation process as well as apoptosis induced by generation and accumulation of ROS. The accumulation of oxidative stress with APR-246, a treatment able to reactivate wtp53 functions, makes mutp53 cells more sensitive to apoptosis cell death. Antagonizing ROS accumulation with N-acetyl cysteine avoids cell death under these conditions (Deben et al., 2018). This evidence highlights the importance of REDOX balance, since cancer cells, upon losing their antioxidant capacity, sensitize cells to death by ROS accumulation, which can be employed to compromise mutp53 cancer cells leading to apoptosis.

Cordani et al. (2018) observed that ROS accumulation in cells with mutp53 improves mitochondrial membrane potential without affecting mitochondrial DNA. However, the addition of an oxidant stressor such as H_2_O_2_, makes cancer cell lines with different p53 mutants more sensitive to apoptosis. These authors propose that the Achilles heel that compromises mutp53 is based on REDOX balance (Cordani et al., 2020). This process could be employed for the implementation of therapies that allow a decrease in mutp53 GOF through ROS generation.

**Figure 8:**
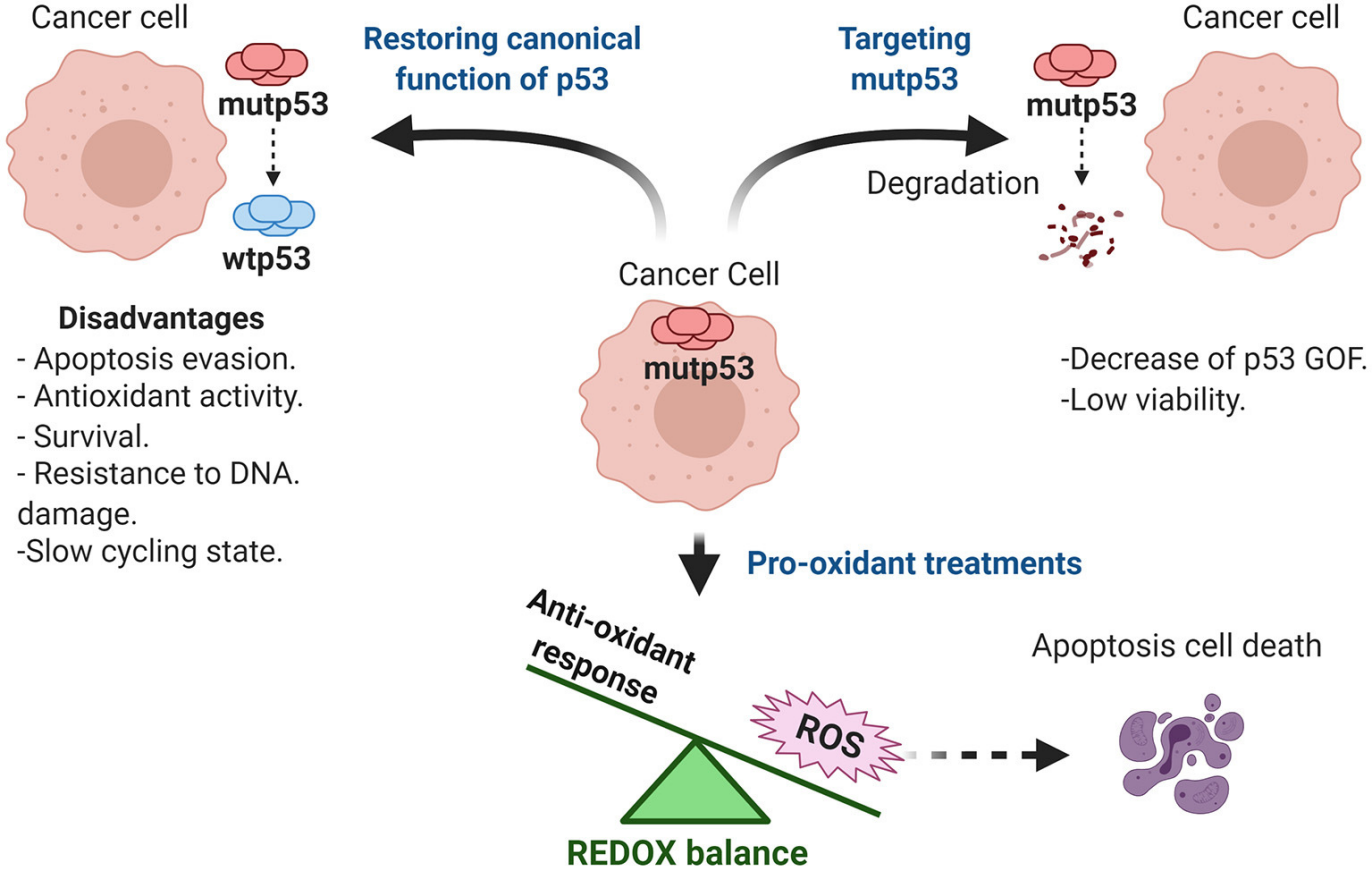
Therapeutic approaches targeting mutp53. Therapeutic strategies for treatment of tumors with mutp53 include reestablishing canonical functions of wild type p53, blocking mutp53 GOF, as well as pro-oxidant treatments.

Additionally, these authors reveal that AMPK inactivation driven by “hotspot” mutp53 can be through different mechanisms, including sestrin inhibition. Sestrins are enzymes that bind directly to AMPK and promote its activation (Cordani et al., 2016). Importantly, AMPK inactivation reduces the activity of PGC-1α and UCP2, both proteins implicated in ROS-scavenging. The pro-oxidant response caused by mutp53 can be used to compromise the functions of mutp53. Therefore, intracellular ROS accumulation can represent an important feature to explain the genomic instability generated by mutp53 (Cordani et al., 2018). However, cancer cells with mutp53 restrict the levels of ROS to avoid the cytotoxic effects caused by an oxidant state, as was recently described. Mutp53 (R273H) increases the expression and activity of antioxidant protein MnSOD (Manganese Superoxide Dismutase) and SIRT3, preventing the harm exacerbated by ROS (Torrens-Mas et al., 2020). This evidence reveals a novel mechanism that confers cancer cells with mutp53 protection to avoid ROS damage. Thus, the cytotoxicity threshold dictated by ROS, which distinguishes between evading or promoting apoptosis, can be useful for therapy, as shown in Figure 8.

Moreover, it has been proposed that the inhibition of antioxidant capacity, along with prooxidant drugs (like doxorubicin or cisplatin), could sensitize cancer cells to apoptosis. However, the therapeutic feasibility of prooxidant drugs in cancer with mutp53 status is not well defined (Kim S. J. et al., 2019).

### Targeting mutp53 Stability to Decrease GOF

An attractive alternative for therapy involves the targeted degradation of mutp53 protein to reduce its half-life (Freed-Pastor et al., 2012). As mentioned earlier, some p53 mutant proteins, in addition to not being functional, generate aggregates. Under hypoxic conditions, autophagy has been shown to promote degradation of such aggregates through the CHIP protein (C terminus of Hsc70-interacting protein), suggesting it can be a key element to selectively reduce mutp53 GOF under hypoxic conditions (Maan and Pati, 2018).

Accumulation of mutp53 proteins can be regulated through inhibition of the MVA pathway. The accumulation of mutp53 is given by their interaction with several HSP (heat shock) proteins. Their inhibition would not only impact signaling pathways that are dependent on lipogenic routes, but also by the stability of mutp53. Using chemical libraries it has been observed that statins decrease viability of cell lines with mutp53, and that this effect is due to the ubiquitination and degradation of mutp53 through the proteasomal pathway (Parrales et al., 2016; Ingallina et al., 2018). The effects of statins on viability of null or *wild type* p53 cell lines were minimal, supporting their potential role as a viable pharmacological strategy for different types of cancer with mutp53 (Chou et al., 2019). The use of statins has been repositioned for use in cancer treatment. For instance, atorvastatin is actually in phase I of different clinical trials. The use of atorvastatin as monotherapy or combined is currently being studied in acute myeloid leukemia and breast cancer with p53 mutations (NCT0356088 and NCT03358017).

Another proposed strategy is based on degradation of p53 HDAC (Histone deacetylase) whose functions are not limited to histones, but also regulate activity of transcription factors, including p53 (Yan et al., 2013). Inhibition of HDAC using SAHA (suberoylanilide hydroxamic acid) impairs the interaction of mutp53 with HSPs, favoring the interaction with Mdm2 and CHIP, and thereby increasing its degradation (Meng et al., 2018).

## Conclusions

Considering the old and new findings related to mutp53 GOF, many of the intracellular pathways of cancer cells can be explained by p53 status. However, there are challenges that need to be answered such as the complete functions of wtp53, the new mechanisms related to GOF, the adaptation of mutp53 to stress conditions, as well as the influence of mutp53 over immune system cells. This makes it difficult to provide easy solutions for a disease that is not completely understood. The translational focus of this exciting field of knowledge in different types of cancer and, the presence of mutp53 proteins is certainly an important and sometimes crucial driving force in the human tumors harboring them. It is noteworthy that mutp53 proteins affect central cellular and tissue processes and systems that include stemness, immune evasion, metabolic control, migration, proliferation, explaining the biology of cancer cells. The understanding of the molecular bases for mutp53 GOF will hopefully allow us to establish common mechanisms for different cancers, not limited to those that harbor mutp53. Currently, the knowledge of p53 status (wild type or mutant proteins) constitutes a crucial factor for the correct use of anti-tumor treatments. This knowledge will allow a better understanding of multiple processes involved in the behavior of cancer cells, including chemoresistance, immune evasion, promotion of stemness, apoptosis resistance, metabolic reprogramming and autophagy, which can help improve current cancer treatments.

## Author Contributions

EA-O and AG-C conceived this review. EA-O and KC-L drafted the initial version with support of JB-R and MS-S who performed the bibliographical search. EO-S reviewed the manuscript and suggested modifications. AG-C supervised, enriched and corrected different versions of the manuscript whose final version was approved by all authors.

## Conflict of Interest

The authors declare that the research was conducted in the absence of any commercial or financial relationships that could be construed as a potential conflict of interest.
